# Demography and movement patterns of a freshwater ciliate: The influence of oxygen availability

**DOI:** 10.1002/ece3.11291

**Published:** 2024-04-23

**Authors:** Victor Brans, Florent Manzi, Staffan Jacob, Nicolas Schtickzelle

**Affiliations:** ^1^ Biodiversity Research Centre, Earth and Life Institute Université catholique de Louvain Louvain‐la‐Neuve Belgium; ^2^ Centre National de la Recherche Scientifique (CNRS) Station d'Ecologie Théorique et Expérimentale (UAR2029) Moulis France

**Keywords:** cell behaviour, hypoxia, plasticity, sit‐and‐wait, *Tetrahymena thermophila*

## Abstract

In freshwater habitats, aerobic animals and microorganisms can react to oxygen deprivation by a series of behavioural and physiological changes, either as a direct consequence of hindered performance or as adaptive responses towards hypoxic conditions. Since oxygen availability can vary throughout the water column, different strategies exist to avoid hypoxia, including that of active ‘flight’ from low‐oxygen sites. Alternatively, some organisms may invest in slower movement, saving energy until conditions return to more favourable levels, which may be described as a ‘sit‐and‐wait’ strategy. Here, we aimed to determine which, if any, of these strategies could be used by the freshwater ciliate *Tetrahymena thermophila* when faced with decreasing levels of oxygen availability in the culture medium. We manipulated oxygen flux into clonal cultures of six strains (i.e. genotypes) and followed their growth kinetics for several weeks using automated image analysis, allowing to precisely quantify changes in density, morphology and movement patterns. Oxygen effects on demography and morphology were comparable across strains: reducing oxygen flux decreased the growth rate and maximal density of experimental cultures, while greatly expanding the duration of their stationary phase. Cells sampled during their exponential growth phase were larger and had a more elongated shape under hypoxic conditions, likely mirroring a shift in resource investment towards individual development rather than frequent divisions. In addition to these general patterns, we found evidence for intraspecific variability in movement responses to oxygen limitation. Some strains showed a reduction in swimming speed, potentially associated with a ‘sit‐and‐wait’ strategy; however, the frequent alteration of movement paths towards more linear trajectories also suggests the existence of an inducible ‘flight response’ in this species. Considering the inherent costs of turns associated with non‐linear movement, such a strategy may allow ciliates to escape suboptimal environments at a low energetic cost.

## Graphical abstract

Experimental cultures of *Tetrahymena thermophila* were raised under varying degrees of oxygen flux entering the medium. Populations with limited access to oxygen showed slower growth kinetics, characterized by lower growth rates and maximal densities, but persisted longer in time. Some genotypes displayed a higher linearity of movement while other decreased their speed under limited oxygen, which may correspond to typical strategies of ‘active flight’ and ‘sit‐and‐wait’ respectively.

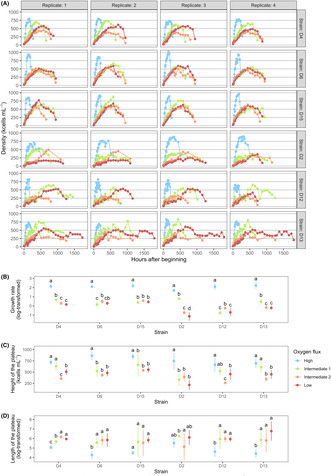

## 1 INTRODUCTION

Oxygen is an essential resource required to sustain metabolic and physiological activity, due to its involvement in the respiratory chains of most prokaryotic and eukaryotic cells (Chance & Williams, 1956; Ingledew & Poole, 1984). In aquatic environments, bioavailable oxygen is present as a dissolved molecule, with varying concentrations and diffusion rates depending on other environmental variables such as temperature, pressure and hydrometric conditions (He et al., 2011; Tromans, 1998). Anthropogenic shifts such as eutrophication and climate warming are predicted to drive considerable changes in the oxygenation conditions of both marine and freshwater bodies. Indeed, recent predictions based on long‐term in situ analyses have revealed that declining dissolved oxygen concentrations (DOC) is a widespread phenomenon in both surface and deep‐water habitats, with main causes ranging from reduced solubility under warmer temperatures, increased phytoplankton production, reinforced thermal stratification and loss of water clarity (Jane et al., 2021). Being subjected to greater fluctuations in oxygen and temperature, freshwater environments are more at risk than coastal or ocean waters, with long‐term declines in DOC up to nine times greater than in the latter habitats (Jane et al., 2021; Knoll et al., 2018). In addition, rising temperatures may decrease the ability of flowing water bodies to ensure the oxygen‐demanding process of waste assimilation, adding to the damage already incurred by a reduction in saturation alone, which should impact high‐velocity streams in particular (Chapra et al., 2021).

Some aquatic species have evolved remarkable adaptations enabling their survival in low oxygen conditions; these include the ability to hyperventilate among water‐breathing ectotherms (Ern & Esbaugh, 2016; Pan et al., 2019), a hypoxia‐induced switch to hypothermia in freshwater crayfish (Dupré & Wood, 1988), the excretion of ethanol by the crucian carp *Carassius carassius* (Vornanen et al., 2009), as well as respiratory proteins with a high affinity for O_2_ and large mass‐specific surface areas of their respiratory organs (Mandic et al., 2009). For organisms that are less tolerant to hypoxia, however, oxygen limitation can entail notable modifications in their overall biology: abrupt variations in DOC can induce morphological changes in yeast (Bellou et al., 2014), modulate the respiration rate of bacteria (Harrison & Pirt, 1967) and drive considerable changes in the physiology and metabolism of fish (Mallekh & Lagardère, 2002; Tran‐Duy et al., 2012). When hypoxic conditions are spatially localized in aquatic environments—for example, minimum oxygen levels are found near the bottom of the water column in thermally stratified lakes (Hanazato et al., 1989)—it may be possible for some species to actively ‘flee’ from low‐oxygen zones, by way of inducible movement behaviours. Such shifts in movement and habitat use have been reported in fish (Bejda et al., 1987; Kramer, 1987) and tadpoles (Marian et al., 1980), showing vertical habitat choice and more frequent surfacing which, in the case of the Atlantic Salmon, was suggested as a proactive strategy to avoid hypoxia (Damsgård et al., 2019).

Although seldom used with respect to oxygen availability, context‐dependent shifts in movement patterns are also involved in common foraging strategies exhibited by a number of terrestrial and aquatic organisms (Janetos, 1982). As opposed to ‘flight’ responses, associated with either predator avoidance (McCarthy & Fisher, 2000) or the long‐distance movements performed by active foragers (Pope & Jha, 2018), certain species have been deemed to perform a ‘sit‐and‐wait’ strategy when faced with resource unpredictability. In the latter case, organisms tend to minimize loss of energy by converting to slower movements and more ‘prudent’ foraging patterns (Butler, 2005; Janetos, 1982). For instance, Nadjafzadeh et al. (2016) identified a general preference for ambush over active foraging in White‐tailed Eagles (*Haliaeetus albicilla*); however, individuals were also reported to switch towards a more proactive form of prey acquisition as environmental conditions became harsher in the winter. Similarly, the scorpion *Scorpio maurus palmatus* can show both strategies alternatively in the face of prey unpredictability, shifting from active dispersal during the rainy season to a prolonged period of inactivity during the dry season (Shachak & Brand, 1983).

Similar responses akin to both the ‘flight’ and ‘sit‐and‐wait’ strategies have been described in ciliates of the genus *Tetrahymena* exposed to food‐shortage conditions (Fjerdingstad et al., 2007); however, such observations are yet to be reported in conditions of limited oxygen availability. *Tetrahymena* ciliates can be found in both standing and flowing freshwater bodies (lakes, ponds and streams, usually with submerged or emergent vegetation; Doerder & Brunk, 2012), and tend to aggregate in swarms near the surface of the water column, which is highly exposed to changes in temperature and UV radiation (Shiurba et al., 2006; Winet & Jahn, 1974). Active cell movements may be spatially oriented depending on social cues, resources, salt or temperature (Campana et al., 2022; Chaine et al., 2010; Giannini & Severson, 2015; Jacob et al., 2018, 2019), with some evidence for oxygen‐driven aerotaxis (Koppelhus et al., 1994; Levandowsky & Hauser, 1978). Moreover, it has been observed that morphological and behavioural alterations enhancing their movement ability can be driven by a perceived change in their environment. A very well‐known response, for instance, is that starvation induces the cell's transformation into a so‐called ‘dispersing morph’, displaying changes in morphology which greatly reflect on its movement capacity, allowing for more coordinated propulsion (e.g. elongated shape, production of a long caudal cilium or ‘flagellum’ in some strains; Nelsen, 1978; Collins & Gorovsky, 2005; Junker et al., 2021). Similarly, Bower et al., 2012 found a trend towards greater swimming speed in response to increasing concentrations of copper, which is known to be toxic and induce peroxide formation in *Tetrahymena thermophila* (Gallego et al., 2007). Significantly faster and more linear movements were also observed in both *T. pyriformis* (Koutna et al., 2004) and *T. thermophila* (Shiurba et al., 2006) subjected to infrared radiation, with the latter showing enhanced differentiation into the dispersal morph.

Prior works suggest that *Tetrahymena* can tolerate a wide range of dissolved oxygen concentrations; however, it can only sustain short periods of anoxia and grows optimally under high oxygen tension (Pace & Ireland, 1945; Pace & Lyman, 1947). Shifts in O_2_ tension are known to affect cellular metabolism in *Tetrahymena*, including peroxisomal fat production, glycogenesis and the rate of oxidation in the Krebs cycle (Pace & Ireland, 1945; Raugi et al., 1975). Elevated oxygen concentrations have been shown to support both steeper growth rates and higher carrying capacities in *Tetrahymena* (Malecki et al., 1971; Pace & Ireland, 1945) and survival of experimental cultures at very low density depends partly on oxygen availability (Christensen et al., 2001). By contrast, very little is known about the putative influence of oxygen availability on inducible cell movements in *Tetrahymena*. As dissolved oxygen concentration appears to be a driving factor for growth in this genus, it has been proposed that such an organism may have developed the ability to detect—and thus adequately respond to—critical changes in oxygen availability (Giaccia et al., 2004; Levandowsky & Hauser, 1978). Moreover, the process of oxidative phosphorylation permitted by aerobic respiration produces 15–20 times more ATP than anaerobic metabolism (Hochachka & Somero, 2002), and movements of individual cilia require the action of dyneins, which hydrolyse ATP molecules (Gibbons & Rowe, 1965; Lodish et al., 2000). We thus expect decreasing oxygen availability to induce changes in the movement patterns of this freshwater ciliate, either as a derivative consequence of reduced performance (e.g. reduced ATP mobilization in locomotory microtubules) or as an inducible response driven by environmental stress, possibly matching either of the aforementioned strategies (i.e. ‘active flight’ vs ‘sit‐and‐wait’).

Here, we exposed six strains of the ciliate *T. thermophila* to four different conditions of oxygen availability (i.e. oxygenated, deoxygenated and two intermediate levels); initial DOCs corresponded to environmentally relevant values ranging from 4 to 9 mg L^−1^, and the rate of oxygen ingress in the culture medium was further manipulated through combinations of shaking, opening and inclining of the culture tubes. We monitored the demography of experimental cultures for up to 1800 h (i.e. several hundred generations), during which we frequently used image and video analysis to record traits of cell morphology (size and shape) and movement (swimming speed, linearity of trajectories and net displacement over a set time duration). We assessed the respective influence of oxygen flux and strain identity on a set of demographic variables (growth rate, height and length of the demographic plateau), as well as the above‐mentioned morphological and movement‐related traits measured during the exponential growth phase. We expected *T. thermophila* populations to adjust their growth kinetics depending on the culture's oxygenation conditions. We also predicted oxygen limitation to influence cell movement, possibly by an increase in speed or linearity indicative of active ‘flight’, as opposed to slower movements characteristic of a ‘sit‐and‐wait’ strategy.

## 2 MATERIALS AND METHODS

### 2.1 Model species, strains and culture conditions

*Tetrahymena thermophila* (Ciliophora: Hymenostomatidae) is a 20–50‐μm long unicellular ciliate found in North American freshwater ponds and streams. As *T. thermophila* reproduces asexually in laboratory conditions, distinct strains (i.e. clonal lineages of cells with an identical genotype) can be isolated and maintained in vitro (Collins & Gorovsky, 2005; Doerder et al., 1995). Here, we used six strains originally collected by F. P. Doerder and provided by the Tetrahymena Stock Centre, identified as D2, D4, D6, D12, D13 and D15. These were chosen from a collection of 22 strains maintained in our laboratory to cover a wide range of characteristics for demography, movement and oxygen consumption (for a detailed characterization of each strain, see Appendix S1). Laboratory stock cultures were kept in sterile multiwell plates (Greiner Bio‐One CELLSTAR®) filled with 2 mL of axenic PPYE growth medium (0.6% Difco Proteose Peptone and 0.06% Difco Yeast Extract), renewed each week and maintained inside a Sanyo MIR‐554 incubator at a constant temperature of 27°C, with a 12:12 light–dark cycle (Pennekamp et al., 2014).

### 2.2 Experimental design

To evaluate the long‐term impact of oxygen deprivation on the demography, morphology and movements of *T. thermophila*, we raised axenic cultures of the six strains in four different conditions corresponding to varying levels of oxygen availability, designated as ‘High Flux’ (HF), ‘Intermediate Flux 1’ (IF1), ‘Intermediate Flux 2’ (IF2) and ‘Low Flux’ (LF); see below. Four replicates per strain were attributed to each oxygenation treatment, for a total of 96 experimental populations (4 conditions × 6 strains × 4 replicates).

### 2.3 Manipulation of oxygen availability

#### 2.3.1 Preparation of culture medium and maintenance conditions

Cultures were maintained under distinct experimental conditions with the aim to manipulate oxygen flux from the air throughout the experiment. Oxygen flux was manipulated by controlling the opening of plastic caps and the agitation of the tubes (using an orbital shaker platform); this was further exacerbated by tilting the tubes at distinct angles, to modify the total area of the air‐medium interface while maintaining equal volume across all treatments (summarized in Table 1; for a detailed description of the conditions attributed to each treatment, see Appendix S2.1). Since differences in oxygen flux would not create immediately distinct availabilities of oxygen, the initial DOC in the culture medium was manipulated as well to generate an expected gradient during the first few hours. This was done by using either (i) a default solution of autoclaved ultrapure water, (ii) autoclaved ultrapure water deoxygenated via nitrogen bubbling or (iii) a 50:50 mix of the two solutions to dilute concentrated PPYE medium. These techniques were used conjointly to approximate a gradient of oxygen availability across the four experimental treatments, aiming to simulate the highest availability in the treatment ‘HF’, limited oxygen availability in ‘LF’ and two intermediate levels in ‘IF1’ and ‘IF2’.

**TABLE 1:** Summary of the techniques used to initiate and maintain four different conditions of oxygen flux throughout the experiment.

Oxygen treatment	‘High Flux’ (HF)	‘Intermediate Flux 1’ (IF1)	‘Intermediate Flux 2’ (IF2)	‘Low Flux’ (LF)
Medium (type of water used)	Normal	50% normal, 50% deoxygenated	50% normal, 50% deoxygenated	Deoxygenated
Tube cap	Half‐turn opened	Closed	Half‐turn opened	Closed
Orbital shaker	Yes (100 rpm)	Yes (100 rpm)	No	No
Tube tilting	Highly tilted (30°)	Tilted (60°)	Tilted (60°)	Vertical (90°)

#### 2.3.2 Validation of the method

**FIGURE 1:**
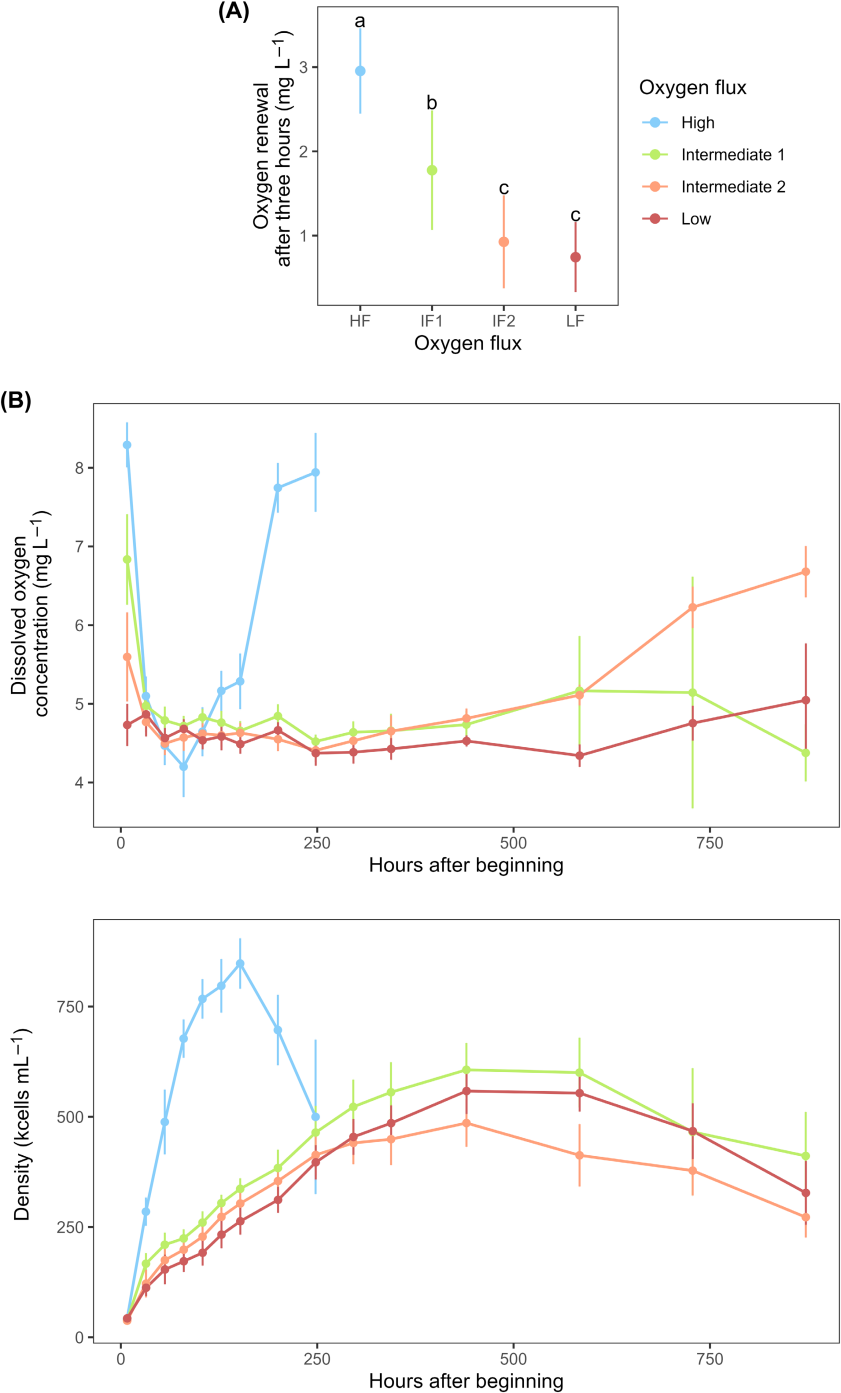
(A) The rate of oxygen renewal is depicted as the absolute difference in DOC between a starting value measured in sterile water immediately after its deoxygenation and a second value measured 3 h later; tubes were kept in conditions identical to the four oxygen treatments, except that no cells of *Tetrahymena* were present during the trial. Coloured dots represent mean values across five replicates and error bars depict 95% CI. Groups sharing a common lowercase letter are not statistically different from each other, as determined from Tukey's HSD test (*α* level = .05). (B) Dissolved oxygen concentration in the medium (mg L^−1^) and cell density (kcells mL^−1^) were measured throughout the experiment, starting from *t* = 8 h. Coloured dots and error bars depict mean values and 95% CI across four replicates of the strains D4, D6 and D15 (individual data are depicted in Appendix S2). Only the growth phases and plateau phases are depicted.

To verify that sensible differences in oxygen flux were generated by our treatments, a complementary trial was conducted in which the increase in DOC was measured in deoxygenated water devoid of *Tetrahymena* cells, after 3 h spent in conditions corresponding to the four oxygenation treatments (the exact protocol is described in Appendix S2.2). This assay confirmed that the rate of oxygen renewal differed significantly across experimental treatments, with the exception of ‘IF2’ and ‘LF’ that showed similar values (Figure 1A). In addition, a second validation trial confirmed that gentle culture agitation per se does not influence cell growth, apart from its ability to favour a homogeneous distribution of oxygen provided by an incoming air flux (see Appendix S2.3).

Observing in parallel the temporal dynamics in DOC and cell density data collected throughout the experiment shows that DOC still differed between treatments at the beginning of their exponential growth phase, 8 h after the start of the experiment (Figure 1B). Following this brief initial period, DOC decreased rapidly throughout the growth phase and stabilized around values ranging between 4 and 5.5 mg L^−1^ (from 32 h into the experiment to almost the end of their respective plateau phase; Figure 1C). A key point to interpret such dynamics is that DOC results from oxygen input and output, that is, the degree of oxygen ingress in the medium from the air (manipulated by the treatments) and how much is consumed by cells (depending on their number and metabolism). This explains why similar DOC values were reached across all treatments during this stable phase, despite large discrepancies in cell density (e.g. fourfold difference between ‘HF’ and ‘LF’ at *t* = 128 h): population grew at their maximum rate, limited by the quantity of bioavailable oxygen, which appeared to be fully consumed in all treatments. DOC rose again after populations had started to decline (due to rapid cell death), hence consuming less oxygen provided by the air; the slope of this DOC increase greatly differed across the treatments, being notably steeper in ‘HF’. These observations strongly imply that distinct degrees of oxygen flux were indeed reached in the different conditions, with those fluxes being fully compensated by cell consumption during the majority of their growth and stationary phases. Demographic curves and DOC measurements are provided for all replicates of three strains (D4, D6 and D15) in Appendix S2; the procedure for DOC measurements is described in Section 2.4 below.

### 2.4 Experimental workflow and procedure

Six days prior to the start of the experiment, four pre‐cultures were created for each strain. This was done by adding 200 μL from laboratory stock cultures into 8 mL of PPYE growth medium inside 30 mL culture tubes (Greiner Bio‐One Ref. 201170, Belgium). These were maintained in an incubator at 23°C and allowed to grow for 6 days to reach a sufficient cell density (around 100,000 cells mL^−1^) to initiate experimental populations from cells in their logarithmic growth phase. On experimental day 1, each pre‐culture was used to initiate four experimental populations (one replicate for each of the treatments). Experimental populations were initiated in tubes of 30 mL capacity by adding 1.4 mL of concentrated PPYE growth medium into 12.6 mL of either normal (‘HF’), mixed (‘IF1’ and ‘IF2’) or deoxygenated (‘LF’) ultrapure water, followed by 1 mL of ciliate cells from a pre‐culture. Culture tubes were placed in their respective maintenance conditions inside a Pol‐Eko‐Aparatura ST2 incubator exposed to natural light variations. Experimental populations were kept at 23°C throughout the experiment, to match the temperature of the laboratory and minimize temperature variations during measurements.

Immediately after their initiation, absorbance was measured for all cultures (*t* = 0 h). Due to time constraints, absorbance was measured at every time point throughout the experiment, while the more time consuming measurements (DOC, pictures and videos) were taken every second time point (starting from *t* = 8 h). Similar to Pennekamp (2014), the delay between time points was increased as growth kinetics slowed down (i.e. up to 24 h on day 8 and 72 h on day 15). DOC, picture and video footage, and absorbance were always collected in this order to minimize the influence of handling and opening the culture tubes on oxygen concentration and cell behaviour. Each experimental culture was monitored for several weeks until it showed density values below 50% of its maximum recorded density for two consecutive measurements, at which point it was discarded; the last culture was discarded on day 80.

Oxygen measurements were performed using a PreSens Sensor Dish plate reader with a PreSens Oxodish OD24 plate placed on top (www.presens.de). Thirty minutes before oxygen was measured, 500 μL of demineralized water was poured into the OD24 plate wells to check for the integrity of the measuring sensors. An opaque silicon cover was placed on top of the OD24 plate cover to protect sensors from external light and prevent their degradation. Cultures were handled individually and 500 μL were collected at 1 cm below the surface of the tube to fill wells in the OD24 plate. The plate reader measured oxygen concentration every 15 s for at least 10 min per well; minimum values were retained as the recorded DOC.

Morphology and movements were quantified through image analysis. For this purpose, a 13 μL sample was taken at 1 cm below the culture surface and poured into one chamber of a counting slide (Kima slide, Ref: 301890). One picture and one 20‐s video were captured in dark field microscopy directly after loading the sample, using a Canon EOS 5D Mark II camera linked to a Nikon Eclipse 50i microscope. Cell *size* (surface) and *shape* (major axis divided by the minor axis of a fitted ellipse) were computed for each individual cell as measures of morphology. Parameters of cell movement included *swimming speed* (gross speed across the whole length of the trajectory), *net displacement* (Euclidian distance between the start and final positions) and *trajectory linearity* (net displacement divided by gross displacement) over the first 5 s of each individual trajectory. Movement and morphology traits were computed during the exponential growth phase only, to avoid potentially confounding effects throughout the later phases (e.g. accumulation of metabolic waste and cellular debris, nutrient shortage and differences in cell density during the plateau). The mean value of all cells identified on a picture or video was attributed to each time point. The final value used for graphical representation and statistical analyses corresponds to the arithmetic mean of all time points belonging to the growth phase (delimited as described in Appendix S3).

To collect morphological data, pictures were analysed with an ImageJ macro and SAS software that identifies and distinguishes cells from potential artefacts based on a set of cleaning criteria (including the area, length, width and brightness of white particles detected over a black background); these criteria were optimized and validated on independent pictures with manually identified cells (Pennekamp & Schtickzelle, 2013). Videos were analysed using the R package ‘BEMOVI’, which reconstructs cell movement trajectories and quantifies movement metrics (Pennekamp et al., 2015). To discard artefacts, that is, particles that may appear moving on the video due to liquid motion generated by neighbouring cells, only trajectories that met some quality criteria were used for video analyses (median displacement between two frames ≥2 μm; total net displacement ≥100 μm; cell detected on ≥10% of the frames).

Cell density was quantified by measuring absorbance (optical density at 550 nm) using a Genesys 20 spectrophotometer (Thermo Fisher Scientific). Lids were closed tightly and cells were homogenized by gentle horizontal shaking before taking measurements. Four identical tubes containing 15 mL of sterile PPYE medium were used to calculate the blank; these remained closed throughout the entire experiment and were measured at every time point. Absorbance (OD 550 nm) was converted into cell density using the calibration curve previously designed for this purpose by Pennekamp (2014). Changes in cell density over time were represented graphically to distinguish the growth phase, stationary phase (or ‘plateau’) and decline phase of the demographic curves. For each experimental population, density data was used to compute three demographic traits of interest, namely the *growth rate*, *height of the plateau* and *length of the plateau*. For more information about the raw processing of demographic data, see Appendix S3.

### 2.5 Statistical analyses

Data were analysed using R version 4.1.2 (R Core Team, 2021). Eight parameters of interest including traits of demography, movement and morphology were individually analysed as response variables via a mixed and crossed two‐way ANOVA with *Oxygen* condition as a fixed factor, *Strain* as a random factor and their interaction (random factor), using the ‘EMSanova’ function in the ‘EMSaov’ package (Choe et al., 2017). *Oxygen* was considered a fixed factor because the four levels were created by design. *Strain* was considered a random factor because the six strains used for the study were randomly chosen among a larger set of available strains, not for themselves but as a way to replicate the experiment on several strains and measure the intraspecific response to *Oxygen*. Error terms for *F*‐tests were computed as described in Sokal and Rohlf (1995) for a mixed crossed ANOVA (see also Table 2). *Growth rate* and *plateau length* were log‐transformed for statistical analyses, to improve conditions of homoscedasticity.

**TABLE 2:** Two‐way ANOVA detailing the effects of *Oxygen*, *Strain* and their interaction on several parameters of demography, movement and morphology.

Response variables	Oxygen	Strain	Oxygen × Strain
(a) Demographic traits			
Growth rate (log‐transformed)	*F*_(3,15)_ = 38.42	*F*_(5,72)_ = 77.89	*F*_(15,72)_ = 27.17
	*p* < **.001**	*p* < **.001**	*p* < **.001**
Plateau height	*F*_(3,15)_ = 25.40	*F*_(5,72)_ = 20.28	*F*_(15,72)_ = 5.00
	*p* < **.001**	*p* < **.001**	*p* < **.001**
Plateau length (log‐transformed)	*F*_(3,15)_ = 14.94	*F*_(5,72)_ = 2.06	*F*_(15,72)_ = 2.48
	*p* < **.001**	*p* = .081	*p* = **.006**
(b) Movement traits (growth phase)			
Swimming speed	*F*_(3,15)_ = 2.84	*F*_(5,72)_ = 41.43	*F*_(15,72)_ = 3.01
	*p* = .074	*p* < **.001**	*p* < **.001**
Trajectory linearity	*F*_(3,15)_ = 4.74	*F*_(5,72)_ = 28.05	*F*_(15,72)_ = 5.95
	*p* = **.012**	*p* < **.001**	*p* < **.001**
Net displacement	*F*_(3,15)_ = 0.04	*F*_(5,72)_ = 25.83	*F*_(15,72)_ = 4.62
	*p* = .99	*p* < **.001**	*p* < **.001**
(c) Morphological traits (growth phase)			
Cell size	*F*_(3,15)_ = 18.08	*F*_(5,72)_ = 151.17	*F*_(15,72)_ = 5.36
	*p* < **.001**	*p* < **.001**	*p* < **.001**
Cell shape	*F*_(3,15)_ = 5.17	*F*_(5,72)_ = 133.22	*F*_(15,72)_ = 9.40
	*p* = **.012**	*p* < **.001**	*p* < **.001**

*Note*: *F*‐statistics, degrees of freedom and *p*‐values are reported; significant *p*‐values (≤.05) are highlighted in bold. Note that with *Oxygen* being a fixed factor and *Strain* a random factor, the F‐test for *Oxygen* uses the mean squares of the interaction as the error term; other tests use the mean square of error as the error term. Detailed ANOVA outputs are reported in Appendices S5–S7.

Post hoc tests (Tukey's HSD test, *α* = .05) were computed using the ‘emmeans’ function in the ‘emmeans’ package and the ‘cld’ function in the ‘multcomp’ package (Hothorn et al., 2016), separately for each strain. The respective proportion of variance (*η* ^2^) explained by *Oxygen* condition, *Strain* identity and their interaction was calculated by dividing the sum of square of each factor by the total sums of squares, including their interaction and the residuals (Fritz et al., 2012; Morel‐Journel et al., 2020). Standardized mean differences (SMDs) were computed to estimate effect sizes, using the ‘SMD’ function in the ‘SingleCaseES’ package (Pustejovsky et al., 2022; Taddei et al., 2021).

## 3 RESULTS

### 3.1 Variance partitioning

**FIGURE 2:**
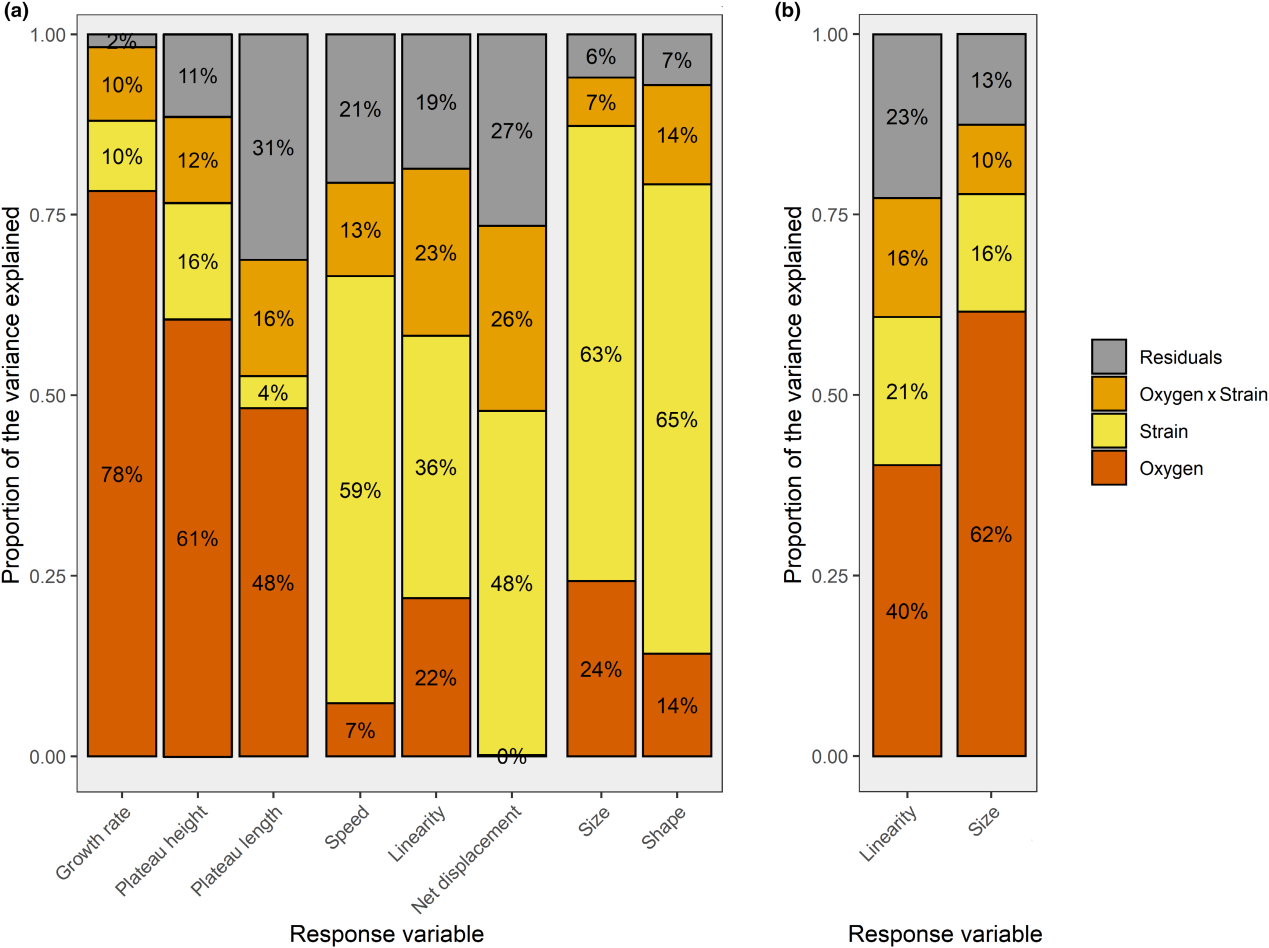
(a) Proportion of variance (*η* ^2^) explained by the factors *Oxygen* (fixed), *Strain* (random) and their interaction (random) on parameters of demography, movement and morphology in *Tetrahymena thermophila*, using a two‐way mixed and crossed ANOVA. Response variables are grouped by demography, movement and morphology respectively. (b) The same analysis was performed by excluding strain D2 from the data set, because its large difference with other strains was responsible for a large part of the total variance, leading to a dilution of the proportion of total variance explained by factors other than *Strain*; response variables shown here (trajectory linearity and cell size) were most strongly affected by the removal of D2.

Variance partitioning based on the ANOVA model allowed us to assess the relative importance of *Oxygen* flux and *Strain* identity on the variance of each trait (Figure 2). All three demographic traits were largely impacted by *Oxygen* flux (up to 78% of variance explained), while variance in morphological traits was rather explained by *Strain* identity (up to 65%). Among movement traits, the main *Oxygen* effect explained a large portion of the variance in trajectory linearity (22%) but little to no variance in swimming speed and net displacement respectively. Instead, the main *Strain* effect and the *Oxygen* × *Strain* interaction were most influential on these two parameters. Strain D2 being much different from other strains for most of the measured traits, this difference was responsible for a large part of the total variance, leading to a risk of perceiving all other sources of variation as negligible (in such an analysis where total variance is scaled to 100%). Variance partitioning was therefore also conducted without this strain; as expected, this led to a notable increase in the proportion of variance explained by *Oxygen* for most traits of interest, particularly for cell size (24%–62%) and trajectory linearity (22%–40%) (Figure 2). For a detailed comparison of variance partitioning including all response variables with and without D2, see also Appendix S4.

### 3.2 Demographic traits

**FIGURE 3:**
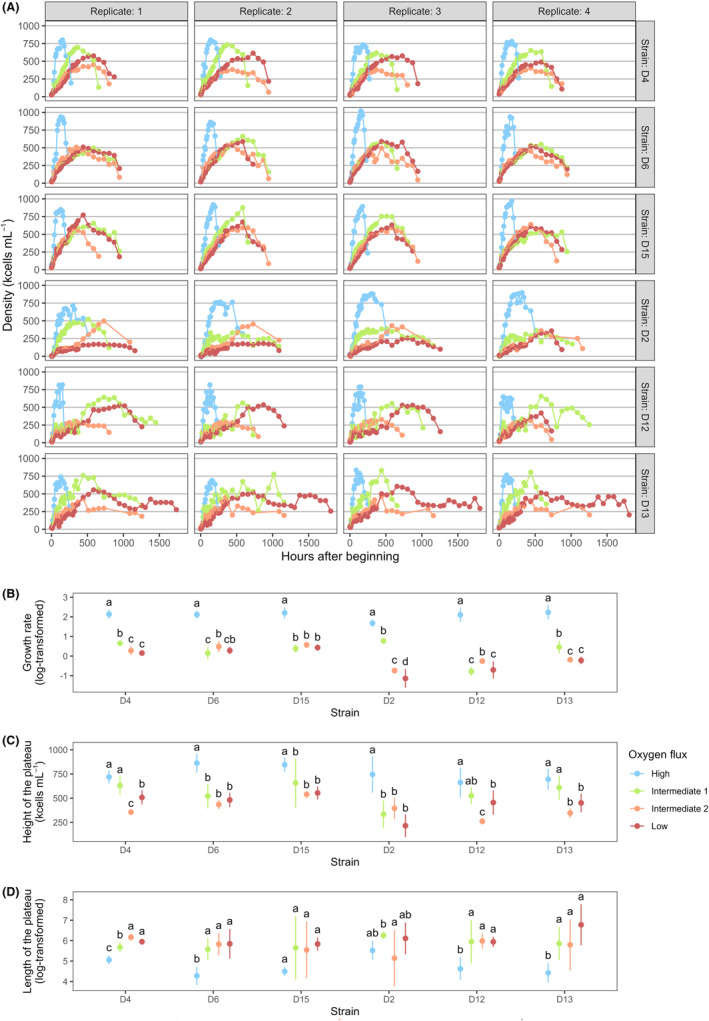
(A) Growth curves of the 96 populations of *T. thermophila* monitored throughout the experiment. Population density over time is depicted for each strain in four different oxygenation conditions. Note that replicates being nested within strain, one replicate of a strain should not be directly compared to the same replicate ID in other strains (i.e. comparing panels within a column is not meaningful). (B–D) Three demographic traits were quantified from these growth curves. Coloured dots represent mean values across four replicates and error bars depict 95% CI. Groups sharing a common lowercase letter are not statistically different from each other, as determined from Tukey's HSD test (*α* = .05) computed separately for each strain.

There was a significant effect of *Strain* identity on two demographic traits (*growth rate* and *height of the plateau*; Table 2) quantified from the growth curves (Figure 3A). The main *Oxygen* effect and *Oxygen* × *Strain* interaction were significant for all three traits: the three treatments with constrained oxygen flux led to a general decrease in growth rate (Figure 3B). A similar decrease was observed for plateau height; treatment ‘IF1’ differed significantly from ‘HF’ in half of the strains (D6, D15 and D2) (Figure 3C). Finally, constrained oxygen flux treatments led to a general increase in plateau length, with the exception of strains D2 and D15 (Figure 3D). Detailed statistical analyses (including sums of squares) are provided for demography in Appendix S5.

Additionally, to better visualize the general direction of oxygen effects on demographic variables, a second analysis was conducted in which those traits were centred by strain, to display the average *Oxygen* effect over all strains. For each replicate of a given strain, the mean of the trait for this strain was subtracted, and the mean across all strains was added. Post hoc tests were performed across treatments, with statistical variance deriving from *Oxygen* condition and biological replication assuming 24 replicates per treatment (Appendix S5).

### 3.3 Movement and morphology

**FIGURE 4:**
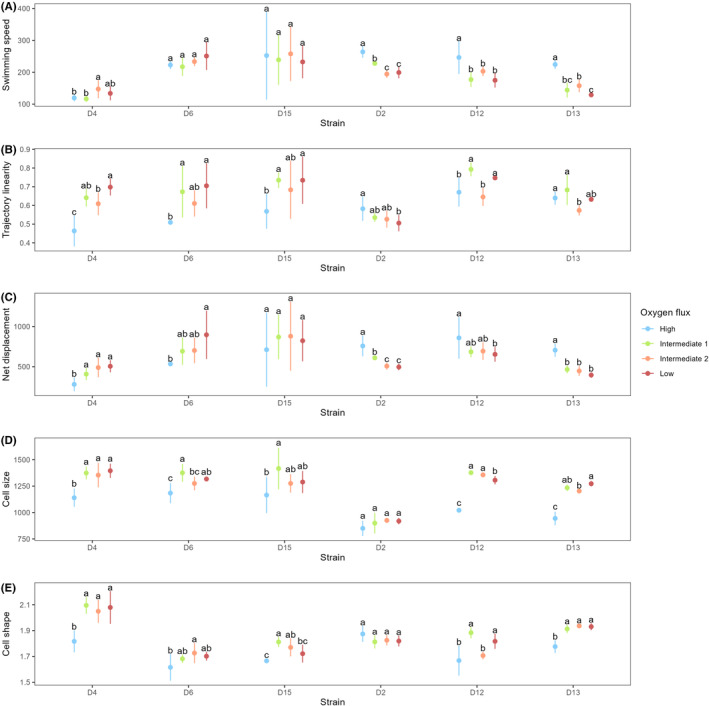
Parameters of movement and morphology quantified during the exponential growth phase. (A) Movement speed (μm s^−1^). (B) Trajectory linearity (higher means ‘straighter’). (C) Net displacement over 5 s (μm). (D) Cell size (μm^2^). (E) Cell shape (i.e. major/minor axes ratio of a fitted ellipse, smaller meaning ‘rounder’). Coloured dots represent mean values across four replicates and error bars depict 95% CI. Groups sharing a common lowercase letter are not statistically different from each other, as determined from Tukey's HSD test (*α* = .05) computed separately for each strain.

There was a heterogeneous response of distinct strains towards constrained oxygen flux (significant *Oxygen* × *Strain* interaction, Table 2). Half of the strains (D2, D12 and D13) showed a significant reduction in their swimming speed between HF and the three other treatments (Figure 4A). Four out of six strains (D4, D6, D15 and D12) showed a significant increase in trajectory linearity between the HF treatment and the IF1/LF treatments; the IF2 treatment was significantly higher than HF only in strain D4 (Figure 4B). As strains D4 and D6 showed an increase in linearity but no reduction in swimming speed, those two strains achieved a greater net displacement over 5 s under conditions of limited oxygen availability. As a consequence of reduced swimming speed, strains D2 and D13 displayed a lower displacement in those treatments. Strain D15 showed no significant change in either swimming speed or net displacement. Effect sizes were considered large for all of the aforementioned comparisons between HF and LF, as depicted in Appendix S6; interpretation of SMD values based on Cohen (1988) and Andrade (2020).

Oxygen flux had a significant effect on the size and shape of cells during the growth phase (*p* < .001, Table 2). Specifically, cells raised in HF conditions were both smaller and less elongated than cells raised in the other three treatments (these did not differ significantly from each other; Figure 4D,E). Detailed statistical analyses (including sums of squares) are provided for movement and morphology in Appendices S6 and S7.

## 4 DISCUSSION

### 4.1 Effects of oxygen availability on population demography: Evidence for plasticity in life history

Early interest in the effects of culture aeration and oxygen tension on the growth of protozoan can be found throughout the twentieth century, including pioneer works on *Tetrahymena geleii* (Pace & Ireland, 1945) and other ciliates (Hall, 1933; Jahn, 1936; Lwoff, 1932). However, results provided by these studies appeared contradictory at times: while Pace and Ireland (1945) reported faster growth of *T. geleii* under increasing oxygen tension, Malecki et al. (1971) found that only the carrying capacities of *T. pyriformis* decreased with reduced oxygen tension, with no detectable impact on growth rates. Here, we collected cell density data monitored for up to 1800 h in the common North American species *T. thermophila*, which included the complete duration of each population's demographic plateau. This allowed us to verify whether limited oxygen flux could constitute a sensible form of stress affecting cell growth (e.g. similar to nutrient starvation; Pennekamp, 2014), thus potentially calling for the expression of behavioural plasticity. We found both steeper growth rates and higher demographic plateaus (i.e. maximal density) in the treatment with enhanced access to oxygen (‘High Flux’). It is worth noting that, assuming normoxic conditions and replete nutrient availability, all six of these strains had previously displayed similarly fast growth and short‐lived stationary phases, which has been described as a ‘boom‐and‐bust’ strategy (Pennekamp, 2014). As such, the shape of the curves observed in the ‘High Flux’ treatments greatly resembled the typical growth kinetics that would be expected from these strains under standard growth conditions; not all genotypes from this species follow such a ‘fast‐paced’ life‐history strategy, however, other strains of *T. thermophila* have been shown to display a pattern of logistic growth instead (Pennekamp, 2014).

Several hypotheses may relate to the short stationary phase and early decline of populations exposed to the ‘High Flux’ treatment. First, cells provided with higher quantities of oxygen likely had increased metabolic rates, which may have led to a quick shortage of nutrients and oxygen in the culture medium (Almeyda et al., 2015). Metabolism is also considered to be a key determinant of an organism's life‐history productivity, which fits with our observation of decreased growth rates under constrained oxygen availability (Careau et al., 2008; Réale et al., 2010). Second, increased consumption of oxygen also implies elevated production of reactive oxygen species (ROS), which may cause lethal cellular damage (Fenchel & Finlay, 2008). Third, rapid cell death can occur under the effect of intercellular signalling, with cells releasing factors inducing apoptosis‐like programmed cell death (PCD) in neighbouring individuals, a phenomenon that can occur under high population density (Kobayashi & Endoh, 2003); a similar mechanism may have played a part in the rapid decline recorded under high oxygen flux, considering that these populations also reached higher maximum densities than the other treatments.

The altered demographic patterns observed in the ‘Intermediate to Low Flux’ treatments indicate that constrained oxygen flux induced a plastic adjustment of *T. thermophila*'s growth kinetics, which might reflect either strong metabolic constraints on cell division, or the expression of an oxygen‐dependent stress‐coping strategy. These populations grew more slowly and did not reach such high densities as ‘High Flux’; however, they also persisted much longer in time, as evidenced by a shift towards quasi‐logistic growth in those treatments. This third effect was true for all but two strains (D2 and D15), which did not show significant differences in plateau length across all treatments. Strain D2 also showed the strongest difference in growth rate between ‘Intermediate Flux 1’ and the other two hypoxic treatments. Such a difference could be explained by an increased sensitivity of this strain to a homogeneous distribution of oxygen in the medium (e.g. due to preferential positioning in the water column), which should have been favoured by gentle agitation in ‘IF1’ (Hellung‐Larsen & Lyhne, 1992; Brown et al., 2003). Since the agitation test carried out with strain D2 suggested no intrinsic impact of shaking on cultures (Appendix S2.3), its contribution to the bioavailability of dissolved oxygen was likely greater than that of opening the cap in ‘IF2’, explaining the strong difference between these two intermediate treatments.

Interestingly, limited oxygen availability had distinct effects on *T. thermophila* than has been shown for other environmental disturbances. For instance, inducing nutrient starvation in the same six strains of *T. thermophila* led to a steeper decline phase (Pennekamp, 2014). This effect more closely resembles the fast decline experienced by highly oxygenated treatments. As mentioned earlier, this was likely caused by cells reaching the point of nutrient shortage faster than under hypoxic conditions, thus involving the same proximal cause for decline as observed in that prior study. Moreover, nutrient‐starved cells did not increase the length of their stationary phase, as hypoxic conditions were shown to do here. Though it may not be possible to sustain population stability once food becomes limiting, decreasing the availability of dissolved oxygen may in turn trigger a set of life‐history adjustments that contributed to delaying the eventual occurrence of nutrient shortage for a given population.

Considering ongoing climatic changes, natural habitats hosting populations of *Tetrahymena* ciliates are expected to face a combination of elevated temperature and increased fluctuations in the availability of dissolved oxygen (Jane et al., 2021). Incidentally, high temperatures were shown to induce a faster growth rate in *T. thermophila* as well as shorter, albeit lower demographic plateaus (Weber de Melo et al., 2020), which is indicative of decreased carrying capacities. Taken at face value, the opposite effects of warming and deoxygenation on population growth rates could possibly offset each other, thus lessening the impact of either stressor in terms of population dynamics in this species. However, it is more likely that growth rates will be constrained by oxygen as a limiting factor, despite the potential for faster growth induced by warmer waters, making it metabolically impossible for cells to divide faster in environments where oxygen is lacking. More plausibly, additive effects of both stressors could negatively impact the carrying capacity of *T. thermophila* in their natural environment, which may translate to increased risks of stochastic extinction (Desharnais et al., 2006; Griffen & Drake, 2008; May, 2019). Verifying either of those assumptions by following the growth kinetics of *T. thermophila* exposed to simultaneously warmed and deoxygenated conditions could make an interesting avenue as a follow‐up to the present experiment.

### 4.2 Effects of oxygen availability on cell motility: Evidence for altered movement paths

Exposing *T. thermophila* to various degrees of oxygen flux in the culture medium revealed the ability of this species to plastically adjust several life‐history traits, seemingly trading‐off biomass productivity for increased survivability at the population scale. By also investigating morphology and movement traits under the same experimental conditions, we attempted to uncover plastic adjustments of such traits that may relate to either of two coping strategies: the ‘flight response’ or ‘sit‐and‐wait strategy’. Acknowledging that confounding factors may have emerged throughout the stationary phase, such as the accumulation of metabolic waste and cellular debris, nutrient shortage or large differences in cell density during the plateau, morphological and behavioural observations are discussed here with respect to the exponential growth phase only (delimited as described in Appendix S3).

Interestingly, two thirds of the strains used in this experiment significantly increased the linearity of their trajectories under hypoxic conditions (D4, D6, D12 and D15). In the ‘Intermediate Flux 1’ treatment, a 15% increase in linearity allowed strain D12 to compensate its reduction in swimming speed, leading to a net displacement value comparable to that of the ‘High Flux’ treatment. For two strains that did not show a decrease in swimming speed (D4 and D6), following straighter paths resulted in a net spatial displacement that was highest under ‘Low’ and ‘Intermediate Flux’. Such effects suggest the existence of an oxygen‐dependent switch towards straighter movement paths, which might better support active flight responses in this species.

It is generally assumed that animals require extra energy to turn, whether terrestrial, aerial or aquatic (Usherwood et al., 2011). This statement also likely applies to ciliates, due to their active mode of locomotion. Using a combination of modelling and experimental tests on a bird and human model, Wilson et al. (2013) found that the total cost of transport increased with decreasing step length between turns, and that a greater extent of turns during movement also related to higher oxygen consumption. The authors therefore argued that uninformed search strategies should favour constrained turn angles (unless the potential benefits of turning would offset its inherent cost). A secondary advantage of increased linearity lies in the ability of individuals to reach new patches of environments (e.g. local zones with a high‐density in bacterial food or dissolved oxygen). As evidenced by Zollner and Lima (1999), organisms should follow straighter paths when they face greater risks such as dispersing through an inhospitable matrix or displaying limited energy reserves (here with respect to oxygen availability). This advantage of a near‐straight line, highly correlated random walk model over more exhaustive search strategies derives from the fact that linear searching increases the probability of quickly locating favourable patches of environment before death occurs during dispersal. Therefore, such an environment‐sensitive alteration of movement paths towards more linear trajectories may constitute a particularly cost‐efficient (albeit genotype‐specific) solution for spatial escape in *T. thermophila*.

Interestingly, three strains (D2, D12 and D13) reduced their swimming velocity under hypoxia, one of which also displayed the aforementioned increase in linearity. Such a diminution in swimming speed may allow cells to save up on energy expenditure devoted to locomotion and foraging, especially if those movements would not lead to the acquisition of resource in a limited environment. Thus, such strains may rely primarily on a form of ‘sit‐and‐wait response’ when faced with conditions of limited oxygen availability, a strategy that is characterized by prudent foraging patterns and limited movement (Nadjafzadeh et al., 2016; Shachak & Brand, 1983). Although the ‘sit‐and‐wait’ strategy would not permit spatial avoidance of a perceived lack of resource, it is one that revolves instead on a form of temporal avoidance. This may prove especially useful in the face of cyclical or temporary periods of limited dissolved oxygen availability driven by ongoing climatic challenges (i.e. hypoxic episodes), which are predicted to become increasingly more frequent in freshwater habitats (Jane et al., 2021).

Prior to this study, there has been little information as to whether and how decreased oxygen availability could modify the swimming behaviour of *Tetrahymena* ciliates. Koppelhus et al. (1994) found that nutrient‐starved cells of *T. thermophila* did not differ in their swimming speed when placed in either hypoxia or saturated oxygen conditions; it is however unclear whether this observation would apply to non‐starved cells, which may show a broader range of achievable movement speed given a high availability of nutrients, potentially allowing for plastic increases in velocity under hypoxia. Limited evidence also suggests that processes related to ciliogenesis may influence the locomotor ability of *T. thermophila* or *T. pyriformis* exposed to hypoxia or inhalational anaesthetics (Brown et al., 2003; Nunn et al., 1974). Contrary to such prior studies, our assessment of ciliate motility did not revolve around the sole estimation of swimming speed (Koppelhus et al., 1994; see also Kitching, 1939 on *P. caudatum*) or the proportion of active versus stationary cells (Brown et al., 2003); here, the estimation of complementary movement parameters beyond swimming velocity (namely *trajectory linearity* and *net displacement*) was permitted by automated extraction of movement data (Pennekamp et al., 2015; Pennekamp & Schtickzelle, 2013). This allowed us to uncover both additive and compensatory effects of swimming speed and movement linearity on the net distance accomplished by active cell movement under hypoxia.

In accordance with our expectations, a decreasing gradient of oxygen availability did result in altered movement paths in *T. thermophila*; however, the resulting changes in behaviour showed aspects of both the ‘sit‐and‐wait’ strategy (decreased speed of movement) and ‘active flight’ responses (increased linearity of movement, allowing cells to perform increased displacement in space), depending on their genetic identity. Notable observations akin to strategies of active flight were previously found in *T. thermophila* faced with environmental stress or disturbances, either indicated by faster movements (Bower et al., 2012; Koutna et al., 2004; Shiurba et al., 2006), enhanced dispersal rates (Campana et al., 2022; Jacob & Legrand, 2021) or preference‐based habitat choice (Jacob et al., 2018; Laurent et al., 2020). As evidenced here, an inducible switch from erratic movements to potentially slower, more linear trajectories may constitute a particularly cost‐efficient evolutionary adaptation. As to why such a strategy may have evolved: *T. thermophila* mostly live in temperate freshwater ponds or small lakes, where they may experience highly variable conditions. It is known that cells can occupy distinct layers and locations within the water column: for instance, cells may be found closer to the surface, where there is an abundance of reeds and vegetation patches along the margins. Conversely, the bottom of a pond constitutes a particularly efficient site to collect cells from the field, as decaying vegetation and abundant bacteria likely accumulate near the sediment (Doerder & Brunk, 2012; Hersha et al., 2009). Other than nutrient availability, distinct variables such as temperature (Morash et al., 2021), UV radiation (Williamson, 1996) and DOC (Riley & Dodds, 2013; Viet et al., 2016) can be both spatially and temporally localized in the water column, implying, for example, daily and seasonal variability, distance to the surface or shading by bordering vegetation. Such variability in their environment could have plausibly led ciliates to evolve context‐dependent movement responses, such as swimming down to avoid negative stressors when present, while swimming up at other times to improve their chance of finding oxygen.

Since the experiment was not specifically designed to assess strain‐specific divergences in coping mechanisms, but rather aimed to assess general responses to varying oxygen availability given a sufficient level of biological replication (i.e. not limited to a single genotype that might have been peculiar), one can only speculate as to why some strains appeared to favour one avoidance strategy over the other. Nevertheless, it may be that some specific traits which were not assessed in our study tend to coincide with a genotype's ability or choice to perform spatial escape over temporal avoidance. For instance, genotypes that may be less potent in their ability to detect molecules of interest in their surrounding environment (e.g. dissolved oxygen, due to limited aerotaxis) and thus orientate their movement towards specific locations may have been selected to favour ‘waiting in place’ instead of enhancing their locomotor ability. Admittedly, an informative and dedicated comparison among strains of *T. thermophila* would have required performing the experiment on a much larger number of strains, thus allowing to associate their choice of strategy with other life‐history traits (e.g. oxygen consumption, etc). Such an endeavour may be the focus of future studies aiming to further explore those differences using an extended set of strains (as previously seen with Pennekamp et al., 2014).

### 4.3 Linking cell morphology with behavioural plasticity

Both cell size and cell shape strongly differed between the ‘High Flux’ and the ‘Intermediate to Low Flux’ conditions. Cells collected during their exponential growth phase appeared larger and more elongated under hypoxia than under normoxia. This observation goes somewhat against theory, which predicts that smaller cell sizes should emerge as oxygen consumption exceeds oxygen supply (Woods, 1999). This relationship is mostly discussed in the context of elevated water temperature, which is often linked with higher metabolic rates and thus greater oxygen demands. Also described as the temperature‐size rule, this relationship was suggested to reflect a ‘ghost of oxygen‐limitation past’, highlighting that selection may have eliminated genotypes that produced large cells or individuals under warm conditions (Verberk et al., 2021). However, it should be noted that a small size is frequently observed in exponentially growing *Tetrahymena* maintained under optimal rearing conditions (Ormsbee, 1942), including these exact six strains (Pennekamp, 2014). When population growth rate is more so affected than individual development, external conditions may lead to cells dividing at a younger age, and thus being smaller (Pace & Ireland, 1945; Zuo et al., 2012). Conversely, cells from the ‘Intermediate to Low Flux’ treatments likely allocated more resource into individual development, being unable to multiply rapidly due to a lack of oxygen. Their large size and elongated shape also fall in line with results from Hellung‐Larsen and Lyhne (1992), who found that *T. pyriformis* increased in cell volume when cultured in conditions known to inhibit cell division (hypoxia, suboptimal temperature or presence of antimetabolites). Though a round shape may denote distinct forms of stress, including toxicity from ethanol or detergents (Dias et al., 2003; Nilsson, 2005), as well as a possibly abnormal conformation of the cytoskeleton (Williams, 2004), the average shape index of cells raised under ‘High Flux’ still translates to a typical ovoid or droplet shape that can be expected from healthy *Tetrahymena*.

Interestingly, prior work focusing on plastic, motility‐associated changes in cell morphology showed that linear swimming in *T. thermophila* independently correlated with either longer cilia or a narrower cell shape. Although such morphological alterations could have been involved in the plastic increase of trajectory linearity reported here, it should be noted that cilia length was not found to respond to nutrient‐starvation in that study (Junker et al., 2021), begging the question whether this trait may plastically respond to changes in dissolved oxygen availability at all. On the topic of linearity, strain D2 was also distinguishable from other genotypes due to its apparent lack of plasticity in this trait, very little variation in cell size and shape across treatments, as well as a generally lower surface than the rest of the strains (Figure 4). As evidenced by the variance partitioning analysis, this strain alone appeared to have disproportionately contributed to the *Oxygen* × *Strain* interaction and main *Strain* effect. Since D2 differed strongly from other strains for a majority of phenotypic traits, it strongly increased the total variation of the model. This led to a severe underestimation of the main *Oxygen* effect's share in the total variance, particularly for the traits of cell size and trajectory linearity (which were indeed shown to react strongly to oxygen flux in this experiment). Although variance partitioning can provide useful biological information, such an example highlights the risk of introducing strong leverage effects driven by the inclusion of ‘outlier’ genotypes. While using a high number of genotypes constitutes a potential workaround for such analyses, it remains advisable to properly characterize laboratory strains and determine to which extent those could leverage the data, at the risk of modifying biological interpretations.

## 5 CONCLUSIONS

Future anthropogenic changes such as eutrophication or warming have the potential to constrain the availability of dissolved oxygen in freshwater habitats, leading to an increased frequency and duration of hypoxic episodes. This study supports that a decrease in dissolved oxygen concentrations above strictly anoxic conditions has the potential to change a suite of demographic traits associated with the life history of a common freshwater ciliate. Though biomass productivity was seemingly traded‐off in exchange for greater longevity under hypoxia, the associated reduction of carrying capacities may interact with warming to threaten population stability in this species. Unlike the main demographic effects, which were mostly canalized across the six strains, distinct genotypes of *T. thermophila* diverged by which locomotion trait was plastically adjusted under hypoxia. A majority of the strains (D4, D6 and D15) were found to strongly increase the linearity of their trajectory, which could constitute a cost‐efficient boost to strategies of spatial avoidance. Alternatively, other strains (D2 and D13) decreased their speed of movement, which likely reduced their energy demands at the cost of effective travel distance (fitting with the idea of a ‘sit‐and‐wait’ response). Our findings also indicate that both strategies must not be mutually exclusive, as evidenced by the lone strain which simultaneously showed a moderate decrease in speed and important rise in linearity (D12). This suggests that records of cell velocity may not be sufficient to accurately transcribe environmentally driven changes in cell locomotory behaviour, thus warranting the analysis of complementary parameters such as linearity. Future studies may provide further insights into these alternative strategies by identifying possible ‘syndromes’ associated with either spatial or temporal avoidance of oxygen limitation, and investigate the expression of similar responses in other common species of free‐swimming protozoans.

## AUTHOR CONTRIBUTIONS

**Victor Brans:** Conceptualization (lead); data curation (equal); formal analysis (lead); investigation (equal); methodology (lead); project administration (equal); validation (equal); visualization (lead); writing – original draft (supporting); writing – review and editing (supporting). **Florent Manzi:** Data curation (equal), formal analysis (supporting); investigation (equal); methodology (supporting); project administration (equal); validation (equal); visualization (supporting); writing – original draft (lead); writing – review and editing (lead). **Staffan Jacob:** Funding acquisition (supporting); supervision (supporting); writing – review and editing (supporting). **Nicolas Schtickzelle:** Conceptualization (supporting); formal analysis (supporting); funding acquisition (lead); methodology (supporting); project administration (equal); resources; software; supervision (lead); writing – review and editing (supporting).

## CONFLICT OF INTEREST STATEMENT

The authors declare no conflict of interest.

### OPEN RESEARCH BADGES

This article has earned an Open Data badge for making publicly available the digitally‐shareable data necessary to reproduce the reported results. The data is available at [https://doi.org/10.5281/zenodo.7738139].

## Supporting information


Figure S1.

Figure S2.

Figure S3.

Figure S4.

Figure S5.

Figure S6.

Appendix S1.

## Data Availability

The data and scripts supporting this study are available from Zenodo: https://doi.org/10.5281/zenodo.7738139.
