# Supplementary figures and images for: Demography and movement patterns of a freshwater ciliate: The influence of oxygen availability
Source: Ecol Evol. 2024 Apr 23;14(4):e11291. doi: 10.1002/ece3.11291 (PMC11040103; doi:10.1002/ece3.11291)

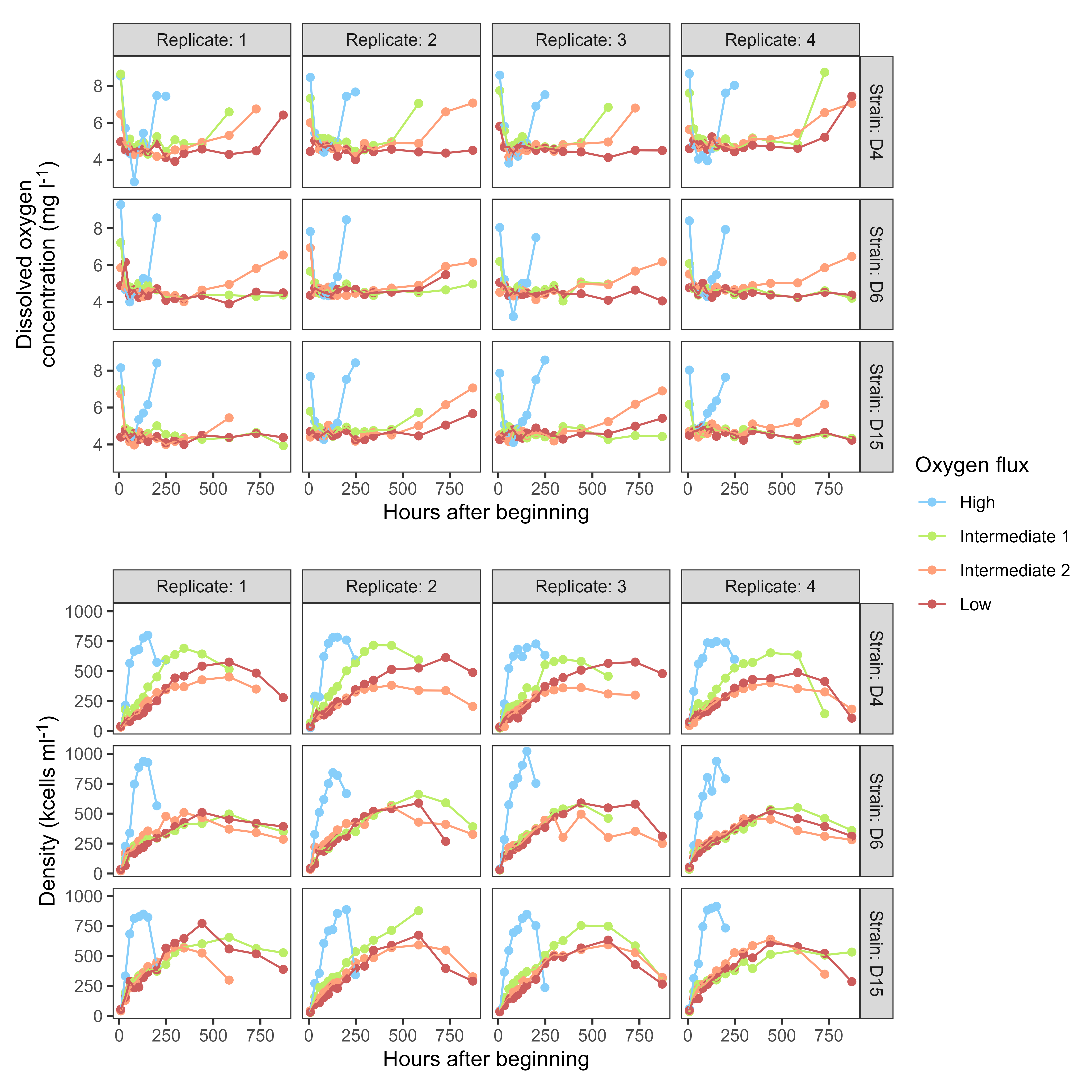

Supplement: Supplementary file 1 — Figure S1. [file ECE3-14-e11291-s004.tiff]

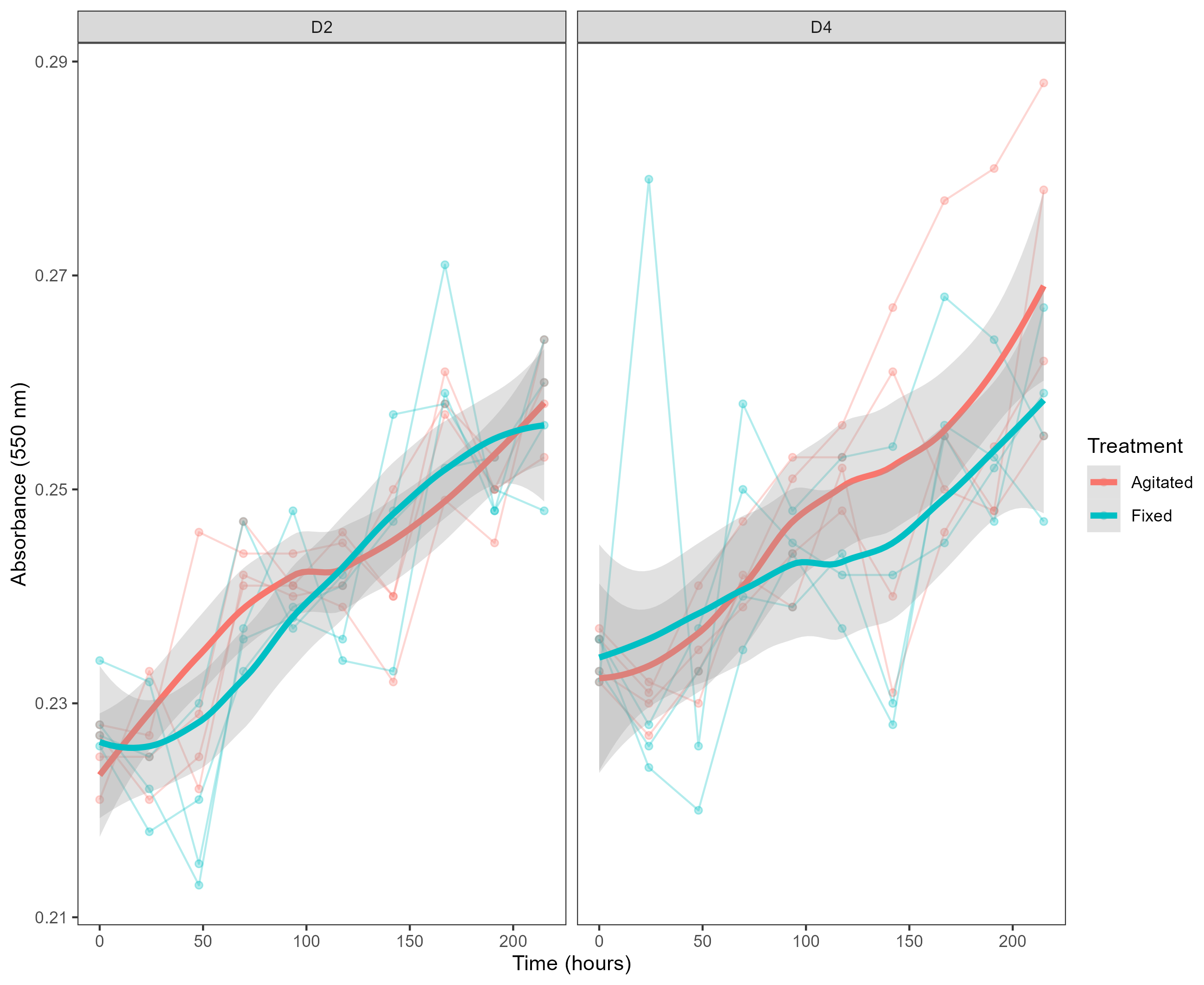

Supplement: Supplementary file 2 — Figure S2. [file ECE3-14-e11291-s007.tiff]

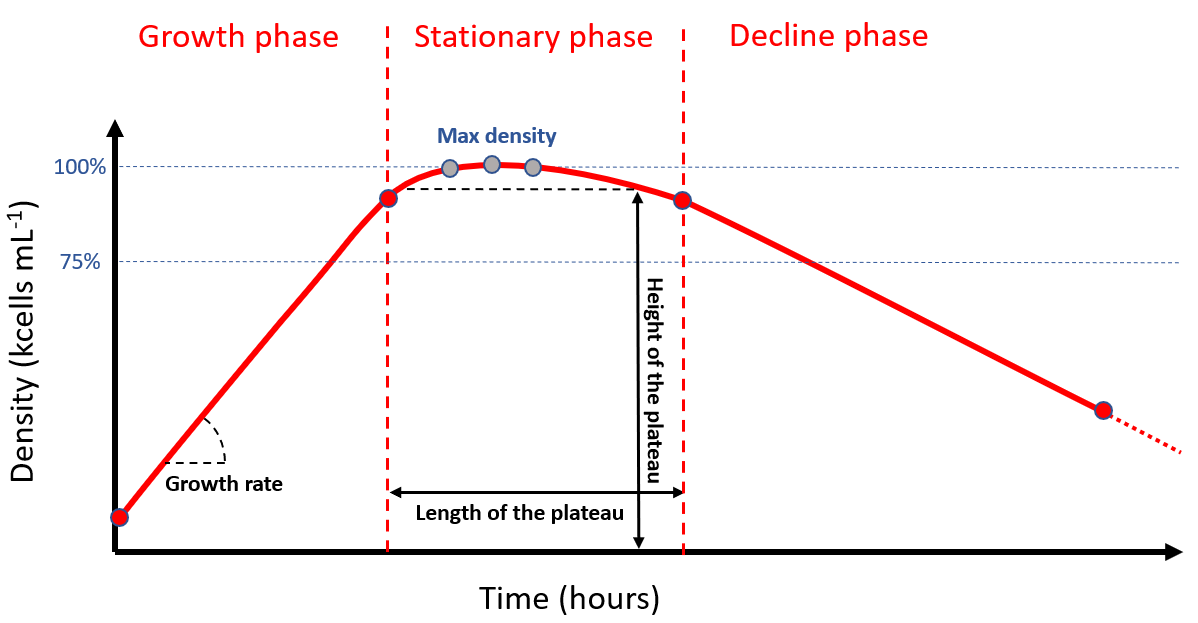

Supplement: Supplementary file 3 — Figure S3. [file ECE3-14-e11291-s006.png]

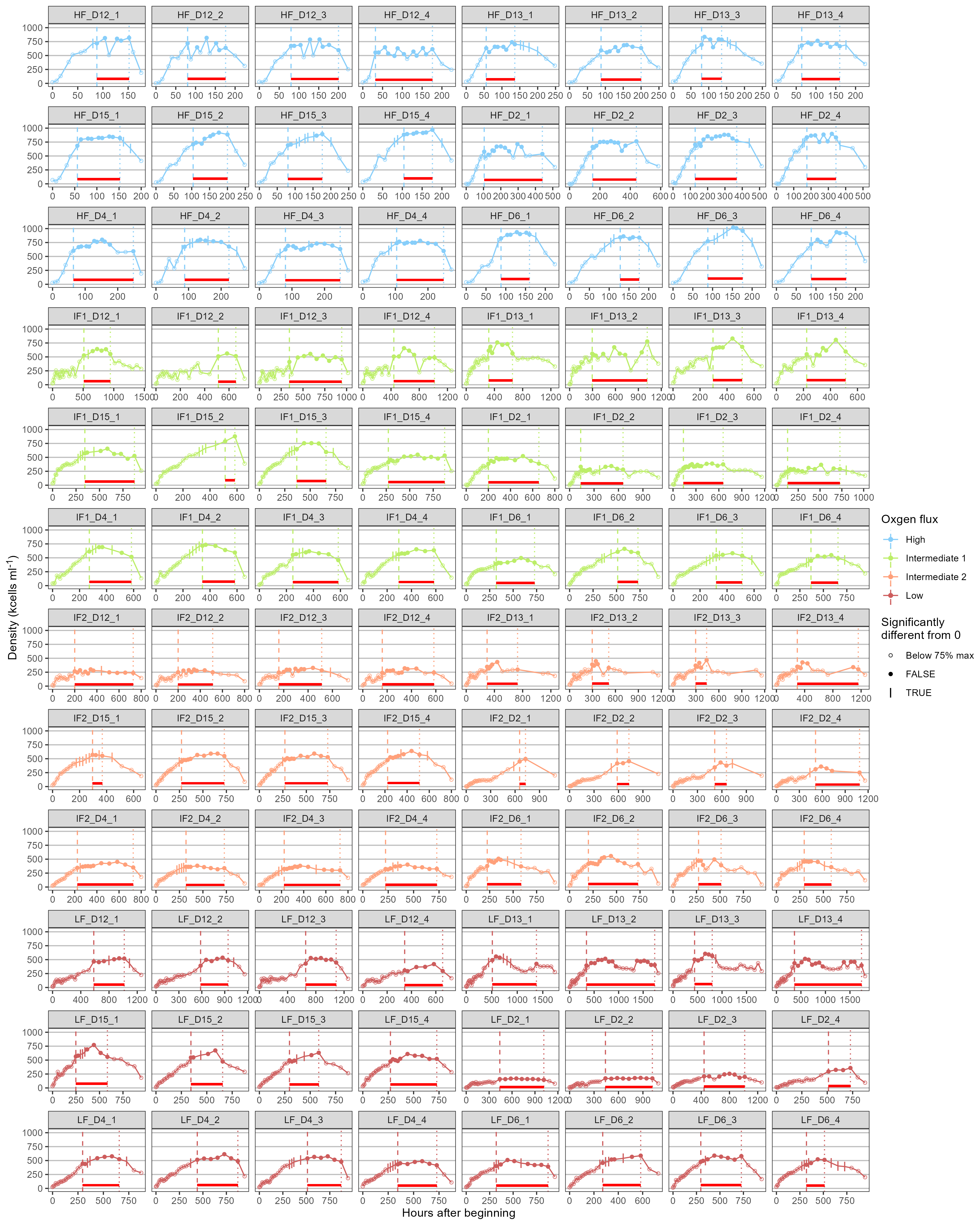

Supplement: Supplementary file 4 — Figure S4. [file ECE3-14-e11291-s005.tiff]

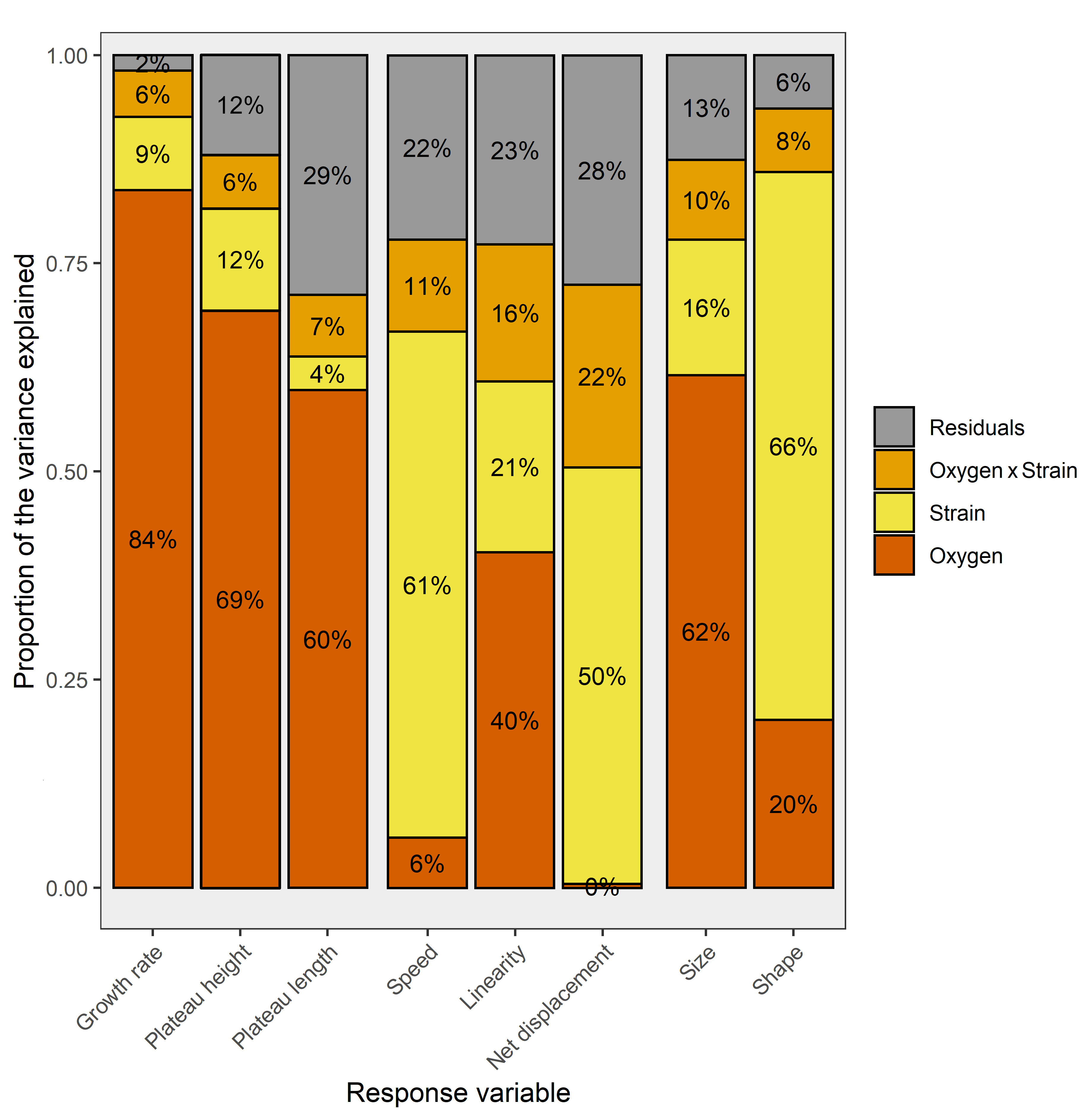

Supplement: Supplementary file 5 — Figure S5. [file ECE3-14-e11291-s002.png]

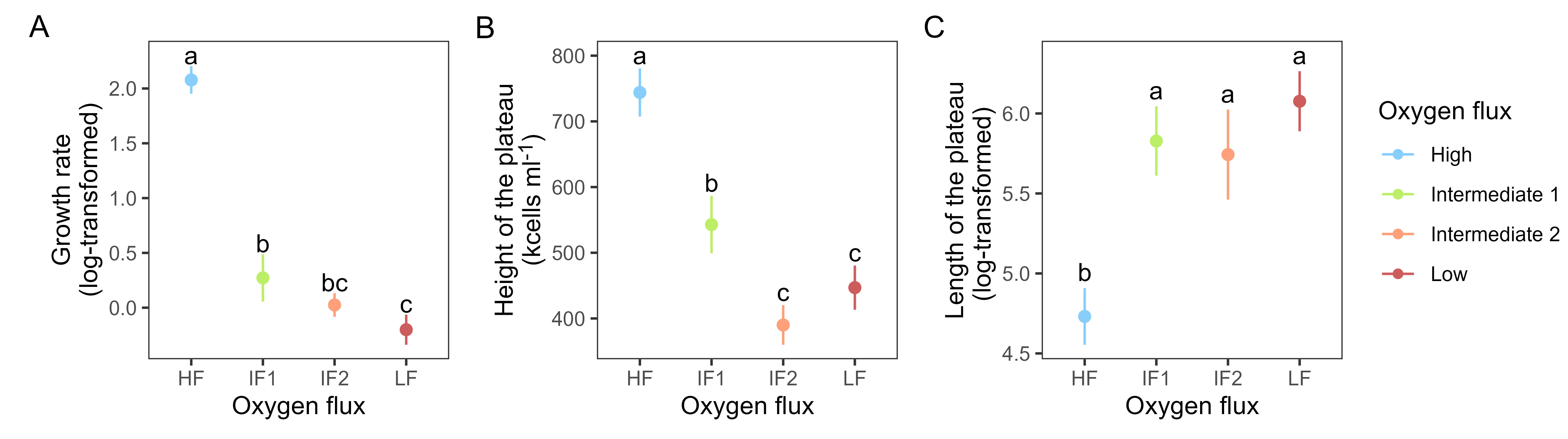

Supplement: Supplementary file 6 — Figure S6. [file ECE3-14-e11291-s003.tif]
