# Supplementary material for: Demography and movement patterns of a freshwater ciliate: The influence of oxygen availability
Source: Ecol Evol. 2024 Apr 23;14(4):e11291. doi: 10.1002/ece3.11291 (PMC11040103; doi:10.1002/ece3.11291)
Supplement: Supplementary file 7 — Appendix S1. [file ECE3-14-e11291-s001.docx]

**APPENDIX S1: CHARACTERISTICS OF THE *TETRAHYMENA THERMOPHILA* GENOTYPES USED IN THIS EXPERIMENT**

**Table S1. Detailed characterisation of the six strains of *Tetrahymena thermophila* used in the experiment.** Information about sample isolation was taken from Pennekamp et al., 2014. Movement characteristics were retrieved from Pennekamp, 2014. Mean activity rate designates the proportion of cells that changed location over three consecutive frames shot at 1s interval. Data for growth kinetics and oxygen consumption at 23°C were collected internally in the research group of N. Schtickzelle at UCLouvain. The maximum relative growth rate per hour (Rmax) corresponds to the intrinsic growth rate of the population when fitting a logistic growth model, calculated as $\frac{ln(\frac{N_{t+1}}{N_{t}})}{\delta_{t}}$, where δt represents an interval of 4 hours.

| **Strain** | **D4** | **D6** | **D15** | **D2** | **D12** | **D13** |
| --- | --- | --- | --- | --- | --- | --- |
| **Reference** | TSC SD01549 | TSC SD01551 | TSC SD01560 | TSC SD01547 | TSC SD01556 | TSC SD01555 |
| **Isolator** | Doerder | Doerder | Doerder | Doerder | Doerder | Doerder |
| **Isolation date** | 06/2003 | 06/2003 | 07/2009 | 08/2002 | 08/2008 | 08/2008 |
| **Geographic location** | Pond SG29, PA | Pond SG29, PA | Gregg Lake, NH | Pond CRWP, PA | Pond SG69-1, PA | Pond SG69-4, PA |
| **Mean speed**  **(µm s^-1^)** | 141.88 | 129.74 | 138.54 | 106.84 | 136.22 | 149.16 |
| **Mean activity**  **rate** | 0.69 | 0.71 | 0.69 | 0.63 | 0.67 | 0.66 |
| **O2 consumption**  **(mg l^-1^ min^-1^)** | 0.19 | 0.10 | 0.13 | 0.16 | 0.25 | 0.11 |
| **Maximum growth rate (R_max_)** | 0.089 | 0.086 | 0.084 | 0.075 | 0.112 | 0.101 |
| **Maximum density (kcells ml^-1^)** | 394.240 | 541.092 | 578.635 | 285.669 | 305.173 | 494.874 |

**APPENDIX S2: MANIPULATION OF OXYGEN FLUX IN THE CULTURE MEDIUM**

**S2.1 Manipulation of oxygen flux in the four experimental treatments**

Oxygen availability was manipulated in two ways: i) experimental populations were initiated in culture media with varying concentrations of dissolved oxygen; ii) culture tubes were later maintained under different conditions to induce varying degrees of oxygen flux into the medium.

*S2.1.i) Oxygen flux*

Plastic caps of the 30 mL culture tubes (Greiner Bio-One Ref. 201170, Belgium) were either i) loosely sealed, unscrewing the lid by half-a-turn (‘HF, IF2’) or tightly sealed (‘LF, IF1’). Loosely sealed caps allowed for oxygen ingress into the tube at all times; tubes were not fully opened to avoid external contaminations (e.g. bacteria) and maintain axenic conditions. By contrast, tightly sealed caps would only allow for very brief periods of oxygen ingress while culture tubes were opened for measurements. In addition, tubes belonging to the same treatment were grouped in plastic racks and either i) held vertically, at an angle of 90° (‘LF’), ii) tilted at an extreme angle of 30° (‘HF’) or iii) tilted at an intermediate angle of 60° (‘IF1, IF2’). For this purpose, tilters were designed and assembled using laser-cut plexiglass. Tilting tubes at a non-perpendicular angle allowed us to increase the area of the air-medium interface, facilitating oxygen ingress (Brown et al., 2003). Finally, tilters supporting the ‘HF’ and ‘IF1’ treatments were placed on top of a Heidolph Unimax 2010 orbital shaker, constantly rotating at 100 rpm. Gentle agitation allows for increased gas exchange at the air-medium interface and facilitates a homogeneous gas distribution inside the tube (Hellung-Larsen & Lyhne, 1992; Brown et al., 2003).

*S2.1.ii) Initial dissolved oxygen concentration*

Cultures from the ‘HF’ (High Flux) treatment were grown with control culture medium, prepared by diluting concentrated PPYE growth medium into autoclaved ultrapure water. Cultures from the ‘LF’ (Low Flux) treatment were initiated in deoxygenated medium. Pure nitrogen in gaseous phase was injected from a pressurised tank into 100 mL of ultrapure autoclaved water with a pressure of 0.1 bar for 20 minutes, under a sterile hood (a similar technique was used previously used in Brown et al., 2003). Concentrated PPYE growth medium was then added to the deoxygenated water; sterile water was deoxygenated prior to adding nutrients to avoid foam formation during nitrogen bubbling. The ‘IF1’ and ‘IF2’ treatments (Intermediate Flux) used growth medium prepared with a 50:50 mix of regular and deoxygenated water, complete with PPYE.

**S2.2 Validation trial: rate of oxygen renewal in the absence of *Tetrahymena* cells**

Prior to conducting the main experiment, a trial was conducted to evaluate the rate of oxygen renewal in the four treatments (‘HF, IF1, IF2, LF’). To this aim, dissolved oxygen concentration (DOC) was measured similarly as described in Section 2.4 of the main text, in deoxygenated water devoid of *T. thermophila* cells. 110 ml of demineralised water was deoxygenated with the technique described above and 25 ml was distributed to four plastic tubes. These were positioned according to each of the four oxygenation treatments, using tilters (‘HF, IF1, IF2’) and the orbital shaker if needed (‘HF, IF1’). DOC was measured in the remaining 10 mL directly after the deoxygenation process, to serve as the reference starting oxygen value. After three hours, DOC was measured in the four tubes. The difference between the reference value and the DOC measured in each of the four tubes was determined as the ‘rate of oxygen renewal after three hours’. This trial was replicated five times; results of multiple comparison are depicted in Figure 1A of the main document, statistical analyses are provided in Table S2.

**Table S2.** One-way ANOVA describing the effect of *Oxygen* conditions on the rate of oxygen renewal after three hours. Significant *P-value*s (≤ 0.05) are highlighted in bold.

|  | Degree of freedom | Sums of squares | Mean square | *F*-value | *P-value* |
| --- | --- | --- | --- | --- | --- |
| Oxygen | 3 | 15.26 | 5.09 | 25.38 | **<0.001** |
| Residuals | 16 | 3.21 | 0.2 |  |  |


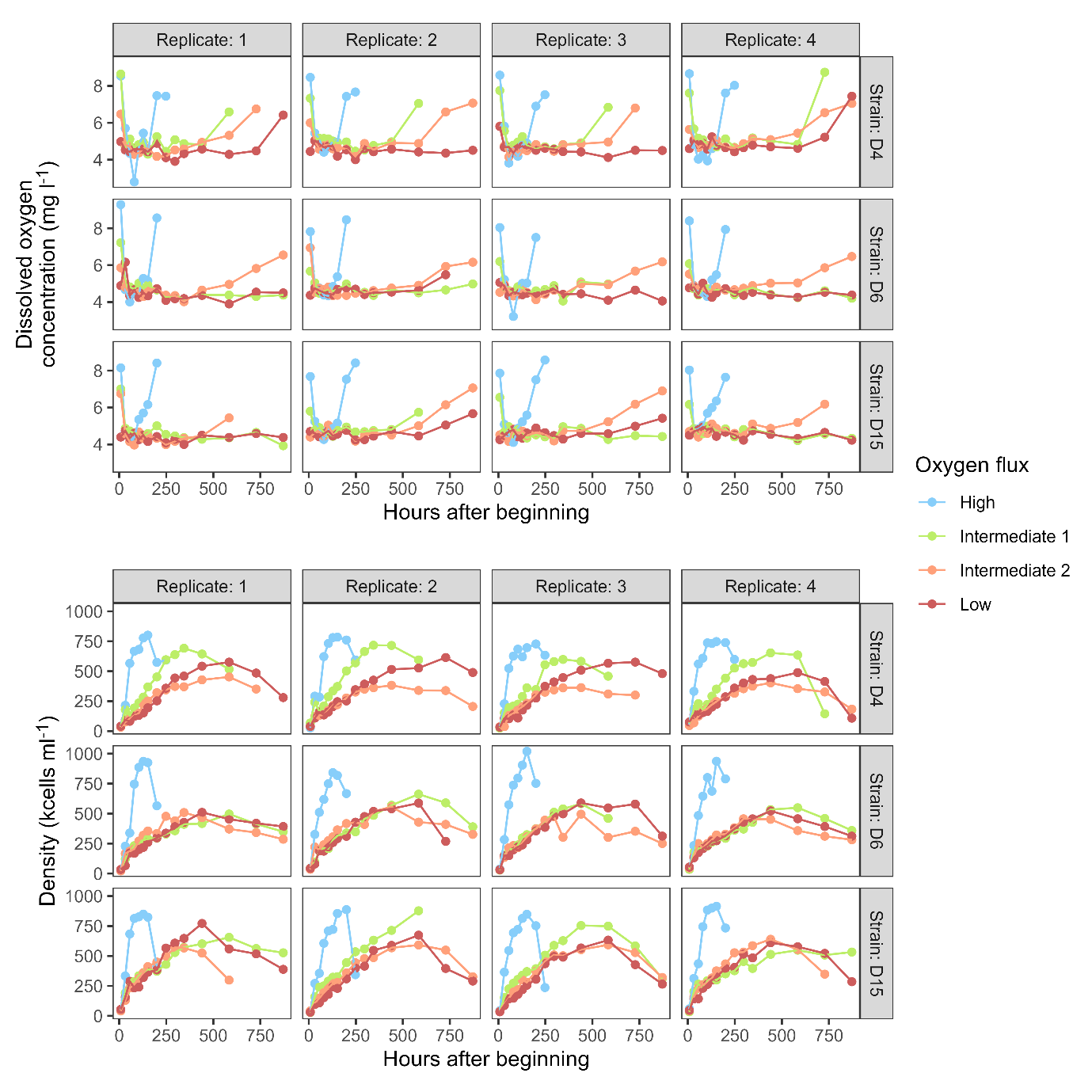


**Figure S1.** Dissolved oxygen concentration (mg l^-1^) and cell density (kcells ml^-1^) were measured throughout the experiment for all cultures of strains D4, D6 and D15 (the first data point was measured at t = 8h). Oxygen concentration in the medium quickly rose as soon as cultures reached the end of their plateau phase.

**S2.3 Validation trial: effect of culture agitation on cell growth**

In the main experiment, constant agitation at 100 rpm was introduced as one of three ways to favour the external flux of oxygen entering the medium in experimental cultures (along with opening plastic caps and tilting tubes at a non-straight angle). Indeed, such a gentle shaking homogenizes the medium, distributing oxygen in all its volume, thereby optimizing its bioavailability to cells and increasing the renewal of oxygen by influx from the air. A secondary trial was conducted to determine whether culture agitation *per se* could have any influence on cell growth, aside from this homogenizing effect. Sixteen tubes (30 ml capacity) were filled with 29.5mL of medium PPYE medium and inoculated with 0.5 mL of either strain D2 (eight tubes) or strain D4 (eight tubes); these two strains were chosen for the trial due to showing significant differences between treatments IF1 (shaken) and IF2 (unshaken) for most demographic traits in the main experiment. All tubes were immediately sealed with fully closed plastic caps; because tubes were filled up to their maximum capacity, there was negligible remaining air between the meniscus and the plastic cap. Tubes were placed on plexiglass tilters at an angle of 30° (the same inclination that was used for the intermediate treatments ‘IF1’ and ‘IF2’ in the main experiment). Absorbance at 550 nm was measured immediately after initiating the cultures; afterwards, four tubes per strain were placed on an orbital shaker rotating at 100 rpm (‘Agitated’), the other four were left unagitated (‘Fixed’).

Absorbance was measured daily and followed for the first 142 hours after inoculation; at this point, all tubes were placed under a sterile hood and left open (cap removed), unagitated, for two hours. This was done to reset oxygen levels in the medium, allowing for cell growth to resume after all cultures had already reached their demographic plateau. After this period, tubes were tightly sealed again, and those belonging to the ‘Agitated’ treatment were placed back on the orbital shaker. Daily measurements of absorbance then resumed for another seven days (168 hours). The resulting data (Figure S2) indicates no discernible effect of culture agitation *per se* at 100 rpm on the growth kinetics of *Tetrahymena thermophila*.


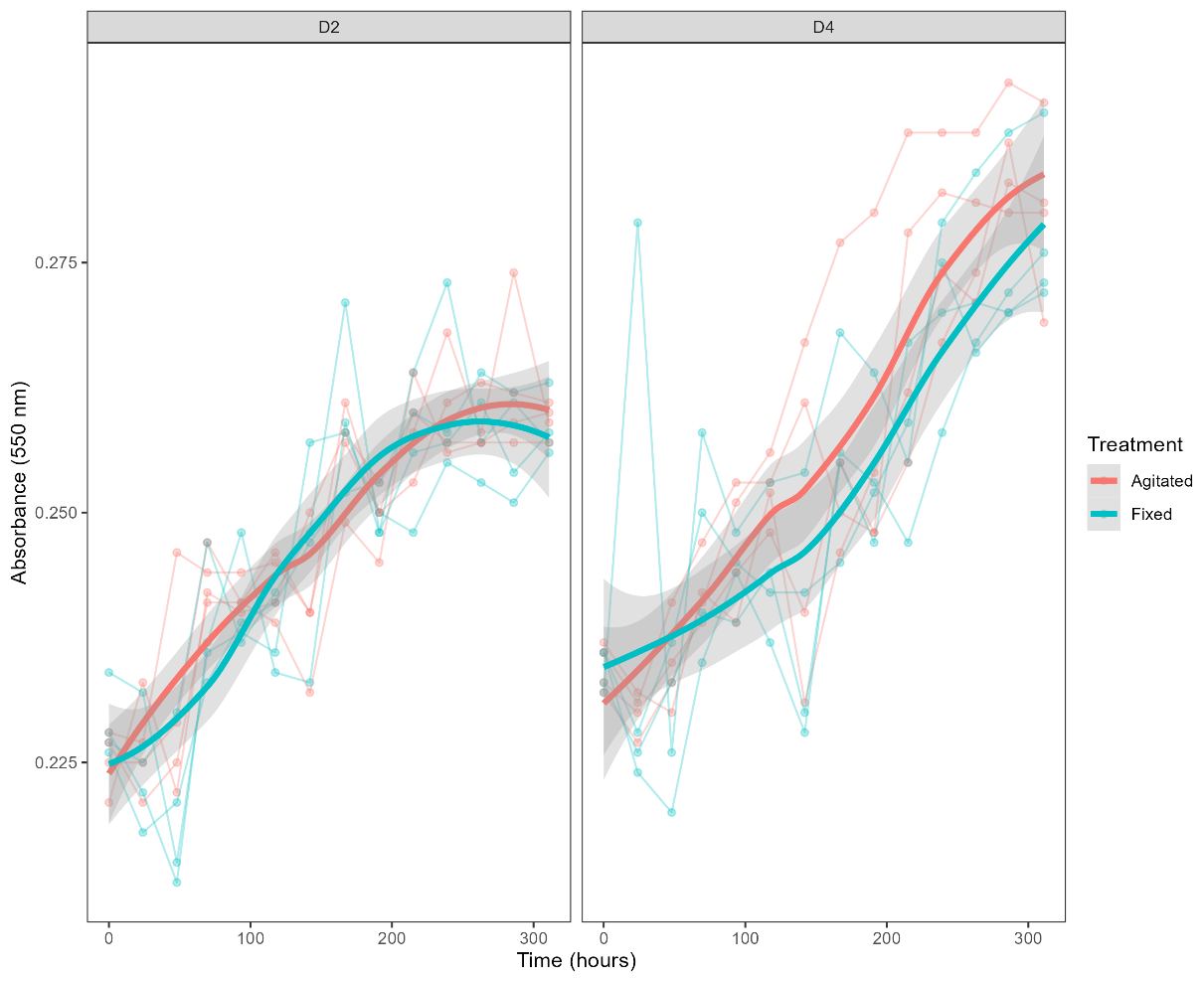


**Figure S2.** Absorbance at 550 nm was measured daily in culture tubes of two strains (D2, D4) filled at maximum capacity with PPYE medium (no remaining air in the tube). Tubes were either ‘agitated’ by an orbital shaker or ‘fixed’ (control). Data smoothing was applied by the LOESS method, grey areas depict 95% CI.

**Table S3.** Two-way ANOVA describing the effect of *Agitation* (fixed), *Strain* (random) and their interaction (random) on the growth rate of *T. thermophila*. Growth rate was defined similarly as in the main experiment. Significant *P*-values (≤ 0.05) are highlighted in bold.

|  | Degree of freedom | Sums of squares | Mean square | *F*-value | *P-value* |
| --- | --- | --- | --- | --- | --- |
| Agitation | 1 | 0.024 | 0.024 | 0.509 | 0.606 |
| Strain | 1 | 0.325 | 0.324 | 12.94 | **0.004** |
| Agitation × Strain | 1 | 0.048 | 0.048 | 1.918 | 0.191 |
| Residuals | 12 | 0.301 | 0.025 |  |  |

**APPENDIX S3: PROCESSING OF RAW DEMOGRAPHIC DATA**


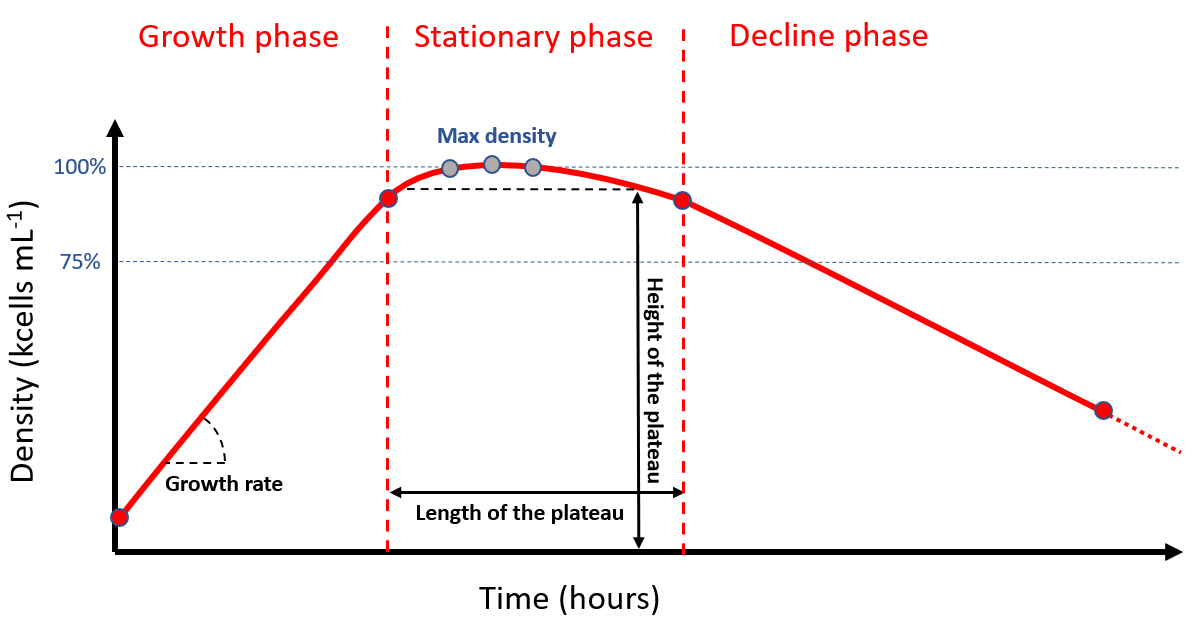


**Figure S3.** Schematic representation of a typical demographic curve showing changes in cell density over time. The three main demographic phases are delimited by red dotted lines. The three demographic traits of interest are indicated in black font. Maximum density (expressed in thousands of cells ml^-1^) was computed as the mean of the three highest density values recorded for each experimental population; this was done to mitigate leverage effects of a single highest density value. The limits between phases were determined by classifying points that belong to the stationary phase (i.e. demographic plateau) as those matching two criteria: density ≥ 75% of maximum density and belonging to a flat section of the curve (i.e. with a slope non significantly different from zero at an α level of 0.01, slope estimated from a linear regression over five consecutive data points: the focal point plus the two prior and the two later points). *Length of the plateau* was defined as the amount of time (in hours) passed between the first and last data point that matched both of those criteria. *Height of the plateau* was computed as the median of all data points included in the plateau phase. *Growth rate* was defined as the slope of the linear regression fitted to the points belonging to the growth phase.


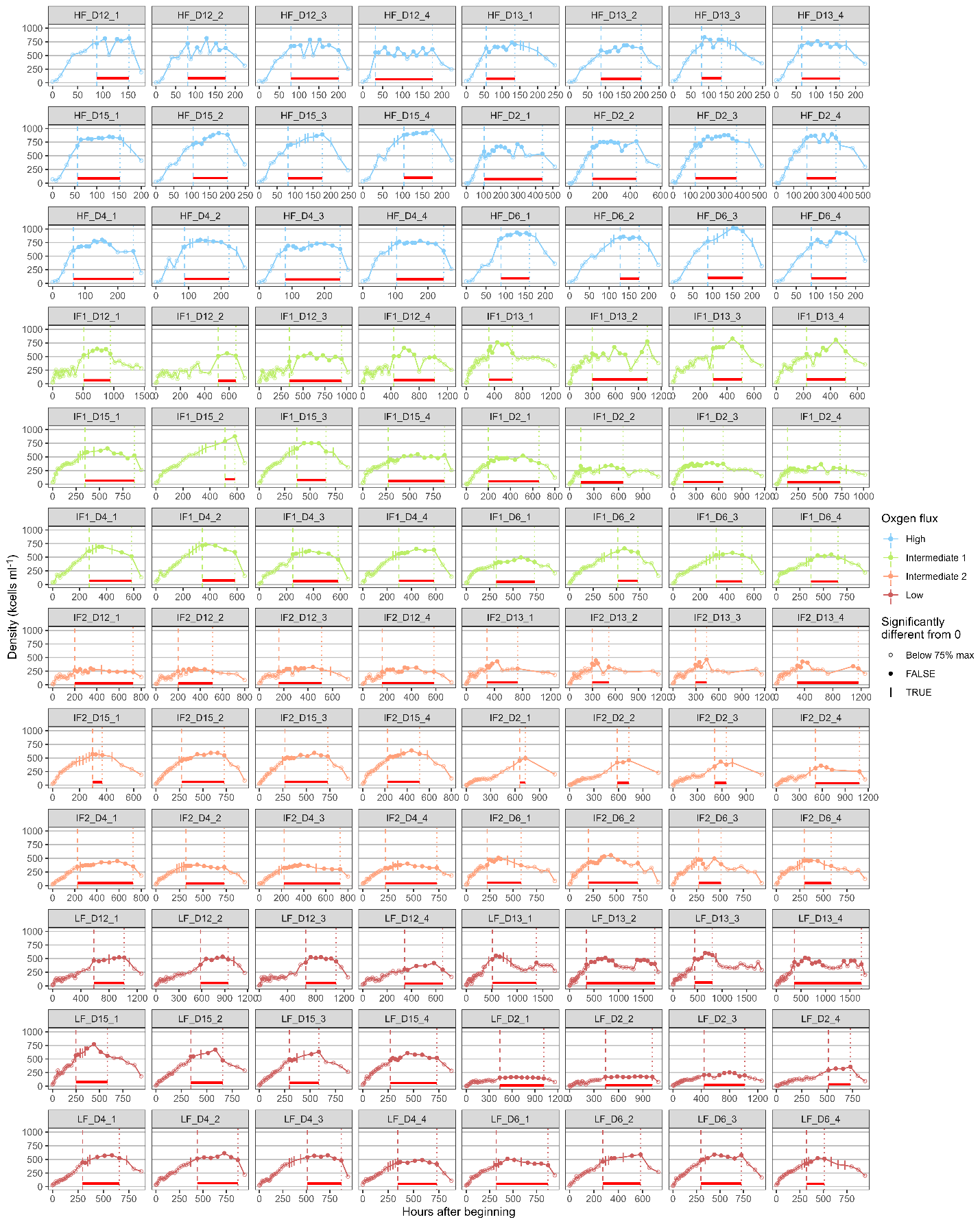


**Figure S4.** Individual demographic curves are depicted for each of the 96 experimental populations, classified by oxygen flux treatment, strain and replicate. Data point symbols indicate the status of the point relative to the two criteria used to define the stationary phase (see Figure S3). The red line below the curve represents the stationary phase, delineated by the first and last points that matched both of those criteria.

**APPENDIX S4: SUPPLEMENTARY RESULTS – VARIANCE PARTITIONING**


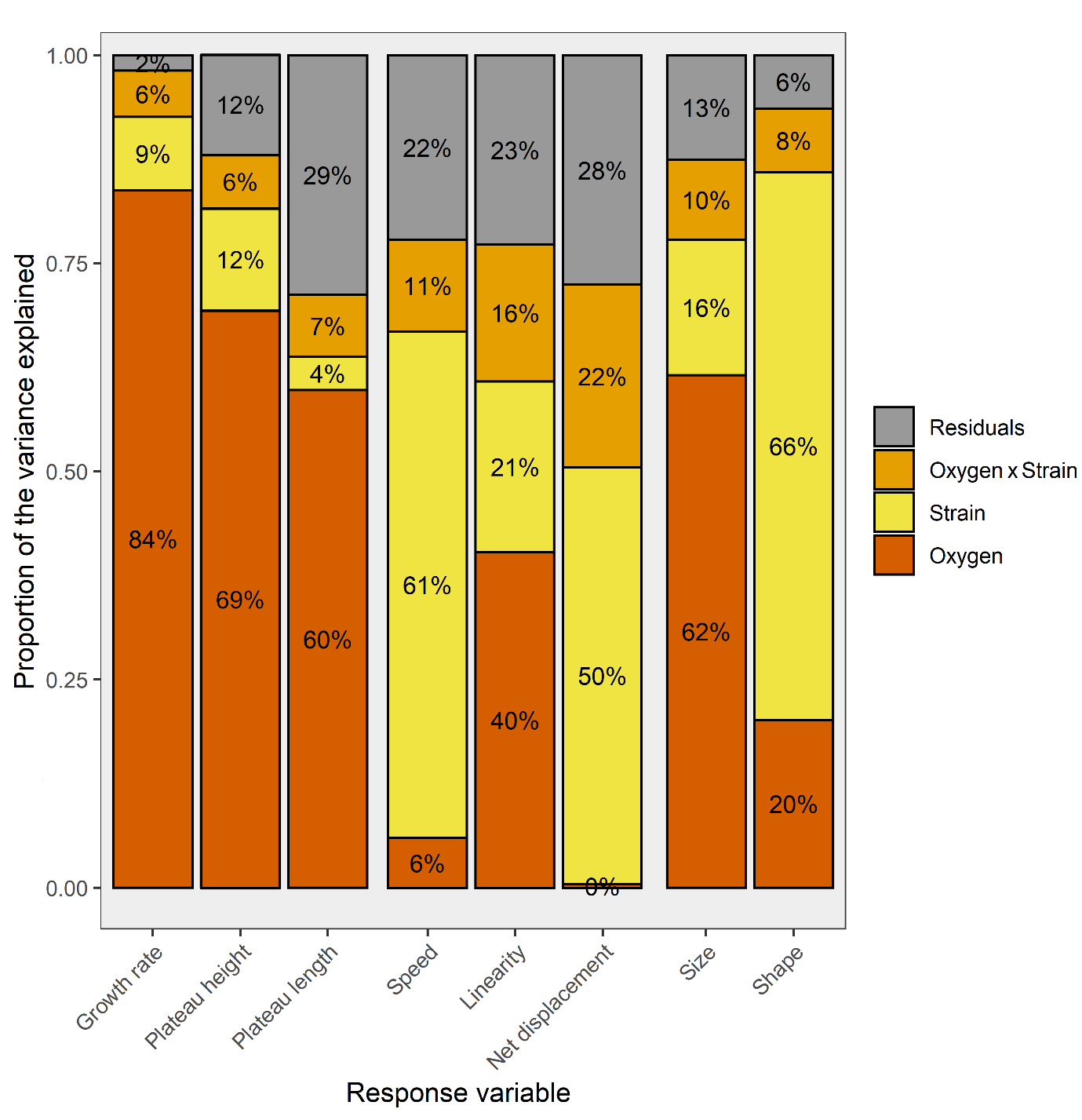


**Figure S5.** Proportion of variance (*η*^2^) explained by the factors *Oxygen*, *Strain* and their interaction using a two-way crossed ANOVA model. Response variables are grouped by demography, movement and morphology, respectively. Compared to Figure 2 found in the main document, strain D2 was removed from the analysis; its large difference with other strains was responsible for a large part of the total variance, leading to a dilution of the proportion of total variance explained by factors other than *Strain.*

**APPENDIX S5: DETAILED CHARACTERISATION OF DEMOGRAPHIC TRAITS**


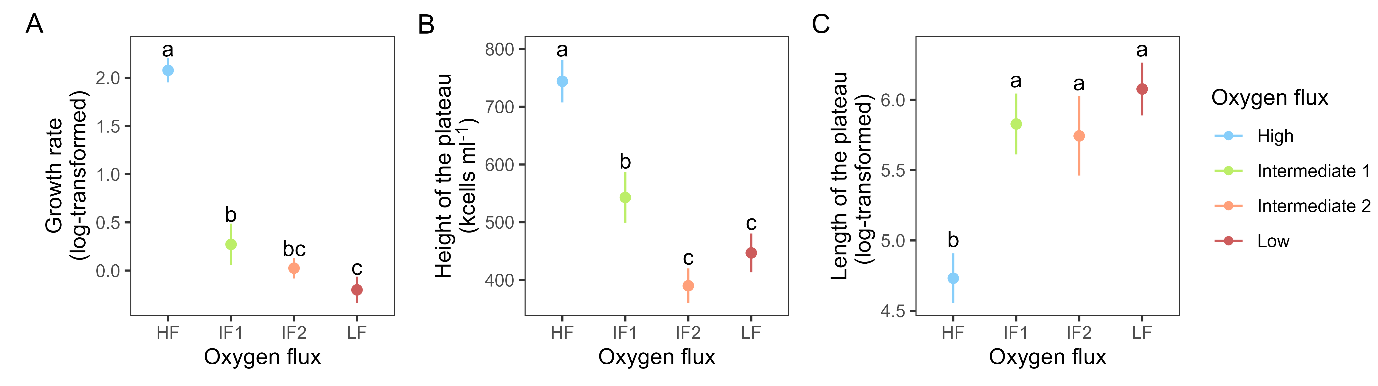


**Figure S6.** Demographic traits of *T. thermophila* compared across the four oxygen conditions. The data were strain-centred to better visualize the main *Oxygen* effect (i.e. the mean of the respective strain was subtracted to each value, before adding the mean of the trait across all strains). **A)** Growth rate (kcells ml^-1^ h^-1^, log-transformed). **B)** Height of the plateau (kcells ml^-1^). **C)** Length of the plateau phase (hours, log-transformed). Coloured dots represent mean values across twenty-four replicates per treatment and error bars depict 95% CI. Groups sharing a common lowercase letter are not statistically different from each other (Tukey’s HSD test; α = 0.05).

**Table S4.** Detailed two-way ANOVA table describing the effects of *Oxygen* flux (fixed effect), *Strain* identity (random effect) and their interaction (random) on the demographic traits: a) growth rate, b) plateau height, c) plateau length. Data was log-transformed for growth rate and plateau length. Significant *P-value*s (≤ 0.05) are highlighted in bold.

| a) Growth rate (log-transformed) | | | | | | |
| --- | --- | --- | --- | --- | --- | --- |
|  | Degree of freedom | | Sums of squares | Mean square | *F*-value | *P-value* |
| Oxygen | 3 | | 78.022 | 26.007 | 38.421 | **<0.0001** |
| Strain | 5 | | 9.704 | 1.941 | 77.892 | **<0.0001** |
| Oxygen × Strain | 15 | | 10.154 | 0.677 | 27.168 | **<0.0001** |
| Residuals | 72 | | 1.794 | 0.025 |  |  |
| b) Plateau height | | | | | | |
| Oxygen | 3 | 1 877 841.8 | | 625 947.27 | 25.399 | **<0.0001** |
| Strain | 5 | 499 404.5 | | 99 880.896 | 20.277 | **<0.0001** |
| Oxygen × Strain | 15 | 369663.8 | | 24 644.254 | 5.003 | **<0.0001** |
| Residuals | 72 | 354 658.2 | | 4 925.809 |  |  |
| c) Plateau length (log-transformed) | | | | | | |
| Oxygen | 3 | 25.3 | | 8.433 | 14.944 | **0.0001** |
| Strain | 5 | 2.343 | | 0.469 | 2.057 | 0.0809 |
| Oxygen × Strain | 15 | 8.465 | | 0.564 | 2.476 | **0.0055** |
| Residuals | 72 | 16.408 | | 0.228 |  |  |

**APPENDIX S6: DETAILED CHARACTERISATION OF MOVEMENT TRAITS**

**Table S5.** Detailed two-way ANOVA describing the effects of *Oxygen* flux (fixed effect), *Strain* identity (random effect) and their interaction (random) on movement traits during the growth phase: a) swimming speed, b) trajectory linearity, c) net displacement over five seconds. Significant *P-value*s (≤ 0.05) are highlighted in bold.

| a) Swimming speed (growth phase) | | | | | |
| --- | --- | --- | --- | --- | --- |
|  | Degree of freedom | Sums of squares | Mean square | *F*-value | *P-value* |
| Oxygen | 3 | 19345.25 | 6448.4174 | 2.8354 | 0.0736 |
| Strain | 5 | 156391.57 | 31278.3137 | 41.4332 | **<0.0001** |
| Oxygen × Strain | 15 | 34113.35 | 2274.2234 | 3.0126 | **<0.001** |
| Residuals | 72 | 54353.54 | 754.9102 |  |  |
| b) Trajectory linearity (growth phase) | | | | | |
| Oxygen | 3 | 0.183 | 0.061 | 4.741 | **0.0161** |
| Strain | 5 | 0.303 | 0.061 | 28.05 | **<0.0001** |
| Oxygen × Strain | 15 | 0.193 | 0.013 | 5.951 | **<0.0001** |
| Residuals | 72 | 0.155 | 0.002 |  |  |
| c) Net displacement (growth phase) | | | | | |
| Oxygen | 3 | 6952.041 | 2317.347 | 0.013 | 0.99 |
| Strain | 5 | 1786879.472 | 357375.894 | 38.062 | **<0.0001** |
| Oxygen × Strain | 15 | 959091.897 | 63939.460 | 7.333 | **<0.0001** |
| Residuals | 72 | 996209.541 | 13836.244 |  |  |

**Table S6.** Effect sizes (95% confidence intervals) testing movement responses to oxygen treatments. The baseline group (‘High Flux’) is compared to the most deoxygenated treatment (‘Low Flux’) for strains that showed either increased linearity or reduced swimming speed under hypoxia (i.e. potential evidence for flight or sit-and-wait responses). Contrasts between oxygen treatments are provided for each strain separately. Standardised mean differences (SMDs) are considered small around absolute values of 0.2-0.5, medium around 0.5-0.8 and high at 0.8 or greater, based on Cohen (1988).

| Response | Strain | Contrast | SMD | CI 95% | Magnitude |
| --- | --- | --- | --- | --- | --- |
| Swimming speed | D2 | High Flux – Low Flux | -4.04 | [-6.59; -1.48] | high |
|  | D12 | High Flux – Low Flux | -1.59 | [-2.80; -0.38] | high |
|  | D13 | High Flux – Low Flux | -8.04 | [-12.80; -3.29] | high |
| Trajectory linearity | D4 | High Flux – Low Flux | 3.25 | [1.19; 5.31] | high |
|  | D6 | High Flux – Low Flux | 17.64 | [5.36; 29.93] | high |
|  | D15 | High Flux – Low Flux | 2.07 | [0.37; 3.77] | high |
|  | D12 | High Flux – Low Flux | 1.15 | [0.17; 2.13] | high |
| Net displacement | D4 | High Flux – Low Flux | 3.06 | [1.04; 5.08] | high |
|  | D6 | High Flux – Low Flux | 22.57 | [5.06; 40.08] | high |
|  | D2 | High Flux – Low Flux | -2.34 | [-3.90; -0.79] | high |
|  | D12 | High Flux – Low Flux | -0.92 | [-1.84; 0.01] | high |
|  | D13 | High Flux – Low Flux | -4.36 | [-7.01; -1.71] | high |

**APPENDIX S7: DETAILED CHARACTERISATION OF MORPHOLOGICAL TRAITS**

**Table S7.** Detailed two-way ANOVA table describing the effects of *Oxygen* flux (fixed effect), *Strain* identity (random effect) and their interaction (random) on morphological traits during the growth phase. Significant *P-value*s (≤ 0.05) are highlighted in bold.

| a) Cell size (growth phase) | | | | | |
| --- | --- | --- | --- | --- | --- |
|  | Degree of freedom | Sums of squares | Mean square | *F*-value | *P-value* |
| Oxygen | 3 | 769 740.2 | 256 580.08 | 18.079 | **<0.0001** |
| Strain | 5 | 2 002 387.5 | 400 477.496 | 151.166 | **<0.0001** |
| Oxygen × Strain | 15 | 212 885.6 | 14 192.373 | 5.357 | **<0.0001** |
| Residuals | 72 | 190 747 | 2649.264 |  |  |
| b) Cell shape (growth phase) | | | | | |
| Oxygen | 3 | 0.241 | 0.08 | 5.165 | **0.0119** |
| Strain | 5 | 1.103 | 0.221 | 133.217 | **<0.0001** |
| Oxygen × Strain | 15 | 0.233 | 0.016 | 9.395 | **<0.0001** |
| Residuals | 72 | 0.119 | 0.002 |  |  |
